# Performance evaluation of enhanced deep learning classifiers for person identification and gender classification

**DOI:** 10.1038/s41598-025-12474-w

**Published:** 2025-08-01

**Authors:** Vasu Krishna Suravarapu, Hemprasad Yashwant Patil

**Affiliations:** School of Electronics Engineering (SENSE), Vellore Institute of Technology, Vellore, Tamil Nadu India

**Keywords:** Person identification, Laplacian transform, Self-spectral attention, Hyperparameter tuning, Gender classification, Periocular biometrics, Computational science, Computer science

## Abstract

Person authentication using periocular images is a prominent research domain. Although the biometric identification systems have advanced, the existing approaches still struggle with accuracy, overfitting issues and computational efficiency, especially when utilizing periocular images for person identification and gender classification. In order to overcome these limitations, this paper proposes an enhanced deep learning classifier (EDLC) paradigm to recognize a person based on the periocular region within a face. A novel Hexagon-shaped ROI extraction is performed in the localization phase to extract the periocular ROIs. Following that, the feature extraction mechanism is accomplished utilizing the Laplacian transform. Finally, three distinct custom EDLCs are employed, such as dilated axial attention convolutional neural network, self-spectral attention-based relational transformer net, parameterized hypercomplex convolutional Siamese network for classification. Further, an adaptive coati optimization algorithm is used to adjust the hyperparameters of the classification models. The efficacy of the model is assessed concerning different outcome indicators. It is also compared with the recent competitive models. For person identification, the SSA-RTNet has achieved a maximum accuracy of 99.8% and 99.67% using the UBIPr and UFPR datasets respectively. Similarly, for gender classification, an accuracy of 98.4% and 99.68% using SSA-RTNet was obtained for the UBIPr and UFPR datasets. As a result, it is perceived that a considerable improvement was observed using the enhanced models.

## Introduction

In the age of biometrics, it can be difficult to identify people and gender uniquely due to the prevalent epidemic^1,2^. Due to the concern about the propagation of infectious diseases, biometric identification facilities based on fingerprints^3,4^ are not contemplated as a secure alternative. Utilizing eye or iris biometrics could be one of the solutions, but this demands a great deal of user cooperation^5^. Periocular infers to the neighborhood surrounding the eyes that includes the eye, brow, and pre-ocular region. Periocular detection utilizes features found in the vicinity of the eye to identify people^6^. One application of the orbital region is the identification of people wearing a mask from a database of photographs^7,8^. Under these circumstances, the facial recognition-based biometric system function becomes ineffective, necessitating the usage of periocular-based face verification^9^. Changes in expression can also reduce the accuracy of facial biometrics^10^. The upper section of the eyes is additionally impervious to divergence in contradiction to the lower portion of the face. It is due to this fact that the periocular identification method has greater expressive power than facial recognition as it includes the top half of the face^11^.

For instance, biometric systems perform well even with deformed faces, but they require a clear view of the eye. The information that the iris and face approaches provide could be supplemented by the periocular modality. Thus, it can be used in conjunction with the iris^12,13^ and face^14,15^ modalities to raise the biometric function’s performance without necessitating changes to the acquisition setup. In the beginning, researchers matched periocular images using manually created features. Descriptors of local as well as global regions are the two primary categories of hand-crafted features. The local feature descriptors split the input image into patches (groups of pels) produce distinct vectors for each patch, and then integrate them^16,17^ to arrive at a particular feature vector. The global feature descriptors, however, deliberate the overall picture and generate one feature vector for the entire imagery. There are three categories into which the global feature descriptors can be further divided: texture-based, color-based, and shape-based attribute descriptors. For matching, various feature descriptions based on texture like local binary pattern (LBP) along with binary-statistical image feature (BSIF) are employed^18^.

The main benefit of texture-based feature descriptors is their ease of use and extremely cheap computational overhead, however, they are also extremely sensitive to image blurriness, noise, and rotation^19^. For matching periocular images, color-based feature descriptors are utilized, and a noticeable identification accuracy is achieved^20^. The main drawback of color-based feature descriptors is that they only function effectively when the fed image has a consistent dispersal of colors. Furthermore, for periocular image comparison, shape-based feature descriptors like the eyebrow shape and the eyelid shape are considered. The major drawback of shape-based features is that they can seldom make it very difficult to discern the contour’s shape from the background color. For example, dark-skinned individuals may find it nearly impossible to extract the eyebrow shape from face images. Local feature descriptors are another type of handcrafted features. With their variations as local feature descriptors, speeded up robust features (SURF) as well as scale invariant feature transform (SIFT)^21^ achieved impressive recognition accuracy. Eventually, it was discovered that the existence of unreal images can impair the system’s comprehensive performance.

Further, in a dual stream convolutional neural network (CNN) with convolutional, FC layer, and a fuse layer with divulged parameters and weights, the disadvantage is its decreased accuracy^22^. On the other hand, when searching for an unknown person, gender information is crucial to generate investigation leads. Numerous applications, including criminology, biometrics, business profiling, and surveillance, use gender classification. However, the operationalization of contemporary gender classification methods remains limited to offense sites given that they depend on the availability of bones, dentition, or dissimilar readily identifiable bodily segments through tangible traits that allow gender determination using conventional methods. Also, the drawbacks of existing biometric techniques comprise their reliance on face or iris traits, which necessitate user cooperation and are impacted by changes in expression or masks. Owing to the issue of limited training capabilities, low accuracy declines image quality, and excessive computing costs. Furthermore, they exhibit overfitting issues and poor performance in identifying fine-grained features.

Few effective techniques for identifying an individual’s gender have already been developed by considering periocular region features. However, there are still some issues, such as high computational costs, deteriorated image quality, increased error, limited training capability, degraded accuracy rate, and a need for large measures of storage space. To address these shortcomings, the proposed work presents an enhanced deep learning classifier (EDLC) paradigm for person identification and gender classification using periocular images. Pre-processing, ROI extraction, feature extraction, and image classification for person identification/gender identification are all parts of the proposed work. Combined tri-lateral guided filtering (CTri-LGF) is used in the pre-processing step to equalize the contrast and embellish the data quality. In the existing works, triangular-shaped and rectangular-shaped ROI extraction methods are employed for ROI extraction. However, they are limited to capturing detailed structure and natural curvature around the eye region. These shapes can miss significant information in curved areas and tend towards a potential decrease in classification accuracy. On the other hand, the hexagon-shaped ROI extraction is more effective since it better conforms to anatomical features of the periocular area, acquiring a more detailed and comprehensive representation. As a consequence, it optimizes the performance of identification and classification. Hence, the ROI in the proposed model is extracted using a hexagon-shaped ROI extraction method. Three EDLCs are further used to learn deep features and perform classification. An adaptive metaheuristic optimization algorithm is used to tune the hyperparameter and minimize loss. Besides, the term enhanced is used to emphasize the improvements made to the standard deep learning classifiers. In the proposed work, specific modifications and optimizations are applied to the baseline deep learning models, such as the consolidation of specialized blocks and tuning the hyper-parameters. These enhancements focus on boosting the classification accuracy, efficiency, and robustness in identifying persons and classifying gender. The following lists the primary contributions of the intended work:

- Designed an inclusive system that addresses the existing issues of biometrics based on deep learning classifiers for two specific tasks such as person identification and gender classification by leveraging periocular images.
- Implemented a hexagon-shaped ROI extraction method that encompasses significant features of the periocular area such as eye shape, eyebrow shape, eye socket, and canthus points and accomplished improved classification performance.
- Utilized three different enhanced classifiers, such as dilated axial attention convolutional neural network (DAA-CNN), self-spectral attention-based relational transformer net (SSA-RTNet), parameterized hypercomplex convolutional Siamese network (PHCSN) in periocular based biometrics for person identification and gender classification. These classifiers are selected for their capability to capture fine-grained features while integrating methods that effectively mitigate overfitting problems, thereby improving generalization performance.
- Employed adaptive coati optimization algorithm (ACoOA) for optimizing the hyperparameters of advanced DAA-CNN, SSA-RTNet, and PHCSN classifiers for the purpose of pursuing high accuracy and reduced errors.
- The remaining structure of the manuscript is outlined in this way: Section “Related works” discusses regarding the relevant works about person identification and gender classification. Section “Proposed methodology” deliberates the envisioned approach. Section “Results and discussion” reveals the outcomes and discussion. Section “Discussion” concludes by discussing the scope of future work.

## Related works

In this section, the recent works done by different authors based on person identification and gender classification are discussed to highlight the key differences. Some of the criteria of prevalent techniques, such as methods, contributions, results, advantages, and disadvantages, are discussed.

### Based on person identification

Utilizing multiple-resolution analysis and local input image features, Kumar et al.^23^ presented a method for periocular identification. Here, wavelet technology was used to do multiresolution image analysis, and then a descriptor with a unique threshold mechanism was used to extract local characteristics from the image. Tiong et al.^24^ recommended employing an RGB-OCLBCP dual stream CNN that could simultaneously process an RGB ocular image and an orthogonal combination-local binary coded pattern (OCLBCP), a color-dependent texture descriptor. By virtue of two separate late-fusionable layers, the contemplated framework combined the RGB image-based OCLBCP descriptors. Pankaj et al.^25^ offered a hybrid CNN beside the gated recurrent unit (GRU) supported framework for face identification. Here, Gabor filtering was used to pre-process the input image. Next, an optimal local mesh ternary pattern (OLMP) was used for pattern extraction. To develop OLMP extraction, the hybrid whale galactic swarm optimization (HWGSO) was employed. Subsequently, the recognition process was carried out using CNN and GRU to improve accuracy. Fadi Boutros et al.^26^ suggested a template-guided knowledge distillation (KD) for periocular face identification. This KD model optimized the distillation process to learn the student model for generating a template comparable to the teacher model.

Bhamare and Patil^27^ demonstrated an automatic person recognition system utilizing periocular visuals built on a blended deep learning model. Images were first pre-processed to enhance noise reduction and image contrast. Mutual conversion swin patch transformer assisted coati depth wise mobile net (MuSwin-Mob) was used to extract the periocular and query image features. The exaggerated archer fish optimization algorithm (ExAFo) was used to choose the relevant features. Lastly, predicated on comparable periocular features, the characteristics of the periocular image and the query were matched using the graph neural network super glue matching algorithm (GNN_SGM) model. Bhamare and Patil^28^ recommended a periocular biometrics-based person identification system using a hybrid optimal dense capsule network (HODCN). The image dimensionality was subsequently minimized by utilizing the 2-D principle component analysis (PCA). Dense convolutional-121 capsule network (DenseCapsNet) was used in the hybrid feature extraction procedure to extract deep features. Through the African vultures optimization (AVO) algorithm, the loss and hyperparameter adjustment were carried out. Weighted distance similarity (WDS) could determine the resemblance amongst the query image and and a collection of images according to the distance score. They have shown significant improvement compared with earlier models. The contributions and constraints of current techniques for person identification are given in Table 1.

**Table 1:** Contribution and limitation of existing methods for person identification.

Author	Approach	Contribution	Result	Advantages	Disadvantages
Kumar et al.^23^	Texture-based constraint	Person identification using wavelets and texture patterns	0.74% of EER	Extracted local characteristics	Need to enhance the performance
Tiong et al.^24^	RGB-OCLBCP DS- CNN	To recognize periocular objects in the environment with improved accuracy	91.28% accuracy	Better accuracy with a large collection of periocular images	Less accuracy and difficulty in identifying people wearing sunglasses or other accessories
Pankaj et al.^25^	Hybrid CNN with GRU	Offer an occlusion-invariant face detection model	96.45% accuracy	Improved accuracy	Need effective localization to further enhance the performance
Boutros et al.^26^	Template-driven KD	Streamlined the distillation process	Error rate of 14.7%	Extracted highly distinctive features	Need to analyze diverse data
Bhamare and Patil^27^	MuSwin-Mob	To offer an automatic person recognition system utilizing pictures of the eyes derived from a hybrid deep learning model	99.58% of accuracy	Maximized the accuracy rate by minimizing the error	High computation complexity
Bhamare and Patil^28^	HODCN	To provide a periocular biometrics-based person identification with maximum accuracy	99.12% of accuracy	Reduced error and high training ability	Need to further increase the accuracy rate

### Based on gender classification (GC)

A periocular classification system that utilizes a CNN-based model and the merging of soft biological attributes with periocular attributes has been proposed by Talreja et al.^29^. The benefit relating to the model was that with the combination of soft biometrics with periocular data and training the entire model, the soft biological attributes increase the network’s ability to discriminate between objects, hence embellishing the network’s competence to do periocular identification. Abdalrady et al.^30^ relied upon the integration of dual convolutional deep-learning Principal Component Analysis networks (PCANet). Using a tiny picture resolution of 48 × 48 pels out of Gallagher’s database, the approach was tested to determine its reliability for gender categorization in unrestricted circumstances. For the specified classification issue, PCANet’s parameters were optimized. Nambiar et al.^31^ offered a deep learning-based EfficientNet framework for determining gender pertinent to periocular visuals. Two methods were used in this, one method was to acquire a CNN-based gender predicting algorithm based on creating a red CNN model, and the other used transfer learning. The model was created from scratch and contrasted with optimized EfficientNetB1. Using periocular images, these models were applied to determine gender and examine the two models based on their respective accuracy. Saeed Aryanmehr and Farsad Zamani Boroujeni^32^ introduced an implementation of iris wavelet scattering for effective deep CNN-based gender classification. The PCA approach was accustomed to minimizing the dimensionality of the collected features and later, the RGB channel’s scattering coefficients were extracted, which were utilized in training the CNN. Furthermore, the attributes derived via the wavelet scattering transform were compared with deep descriptor vectors collected from coarse RGB images. Maryam Eskandari^33^ demonstrated a gender prediction system that combined regional and global facial representations. In order to extract facial features, the BSIF technique was applied to both regional and holistic elements of face images. The optimum subset of characteristics was selected using the particle swarm optimization (PSO) technique. For fuse score levels, the weighted sum (WS) rule method was used. Serin et al.^34^ suggested a CNN-SVM hybrid scheme for gender categorization utilizing fingerprints, which consists of three primary parts: preprocessing, feature extraction, and classification. The primary objective of this method was to extract fingerprint information using CNN. To identify gender, these features were fed into an SVM classifier.

The contribution and constraints of current techniques for gender classification are presented in Table 2.

**Table 2:** Contribution and limitation of existing methods for gender classification (GC).

Author	Approach	Contribution	Result	Advantages	Disadvantages
Talreja et al.^29^	CNN	Soft biometrics-based periocular recognition	97.6% accuracy	Improved network ability. Accuracy enhanced	Need to minimize the overfitting problems
Abdalrady et al.^30^	PCANet	Feature fusion strategy by incorporating two CNN	89.65% accuracy	Reduce the feature vector dimensions	Very less accuracy
Nambiar^31^	Efficient Net	Gender determination from periocular images	97.94% accuracy	The model works ameliorated using standard features	High computational power
Saeed et al.^32^	Efficient CNN	Gender classification using effective feature extraction	92.05% accuracy	Wavelet scattering features were effective	Complexity was more
Maryam Eskandari^33^	BSIF-based gender prediction	To fuse global and regional facial representations for gender classification	The best classification rates of 94.11% and 90.12% for the MBGC and CASIA-Iris-Distance datasets	Improved the performance in terms of different assessment metrics	More complexity, less convergence, and poor classification rate
J. Serin et al.^34^	CNN-SVM	To perform gender categorization from fingerprints	Maximum accuracy of 99.25%	Increase the rate of accuracy	Exhibited overfitting issue

### Based on both person identification and gender classification (GC)

Using the periocular area as a biometric authentication factor, Kumari et al.^35^ offered a reliable solution. For image pairing, the system makes use of both manually produced and automatically crafted features. The model improved in terms of recognition precision for images taken in three unfavorable conditions: subjects putting on spectacles (which can partly block the preocular region), covering of the eye area (effect of limited/full shutting of the eyes), and stance alteration (pictures with inclined heads). Suravarapu and Patil^36^ offered a vision transformer-based model for identifying the person using periocular images. This model was initially pre-trained and scaled the input images to 224 × 224. The stratified k-fold model was applied to avoid overfitting issues. The experimental outcomes demonstrated that the transformer model was effective and appropriate for person identification. Abdullah M. Sheneamer et al.^37^ presented a hybrid person recognition system that combines deep neural networks with machine learning. This method uses deep and machine learning algorithms to identify people based on their gait, whether they are wearing a mask or not, or who cover a significant portion of their face. The endurance and tractability of this approach, which incorporate the foremost aspects of state-of-the-art methods to create a robust and adaptable system, were its primary advantages. The contribution and constraints of current techniques for person identification as well as gender classification are given in Table 3.

**Table 3:** Contributions as well as merits and demerits of existing methods for both person identification and gender classification (GC).

Author	Method	Contribution	Result	Advantages	Disadvantages
Kumari et al.^35^	CNN	To offer a reliable solution for person identification and GC	93.83% and 95% accuracy for person identification and GC	Greater support for feature-level fusion	Limited semantic information and less accuracy
Suravarapu and Patil^36^	Vision transformer	To improve the detection performance	98.18% and 99.13% accuracy for person identification and GC	Highly effective	Complexity is more
Abdullah M. Sheneamer et al.^37^	Deep neural networks with machine learning	To identify people and classify gender at lower computations overhead	99.29% accuracy	Robust and adaptable framework	Not extend the model in unconstrained environments

*Summary:* The identification of persons by automated or semi-automatic means using their physical (iris, facial), behavioral (gait, signature), or biopsychological (EEG, ECG) characteristics, is referred to as biometrics. In recent days, several approaches based on machine learning have been introduced for individual identification. However, the existing methods are devoured with accuracy, more computation time, over-fitting issues, etc. Further, deep learning methods have been introduced for detecting the periocular regions like supervised semantic mask generators and ROI-based object detectors for classifying the individuals. An innovative periocular biometric based on deep learning might be targeted to address these issues.

## Proposed methodology

**Fig. 1:**
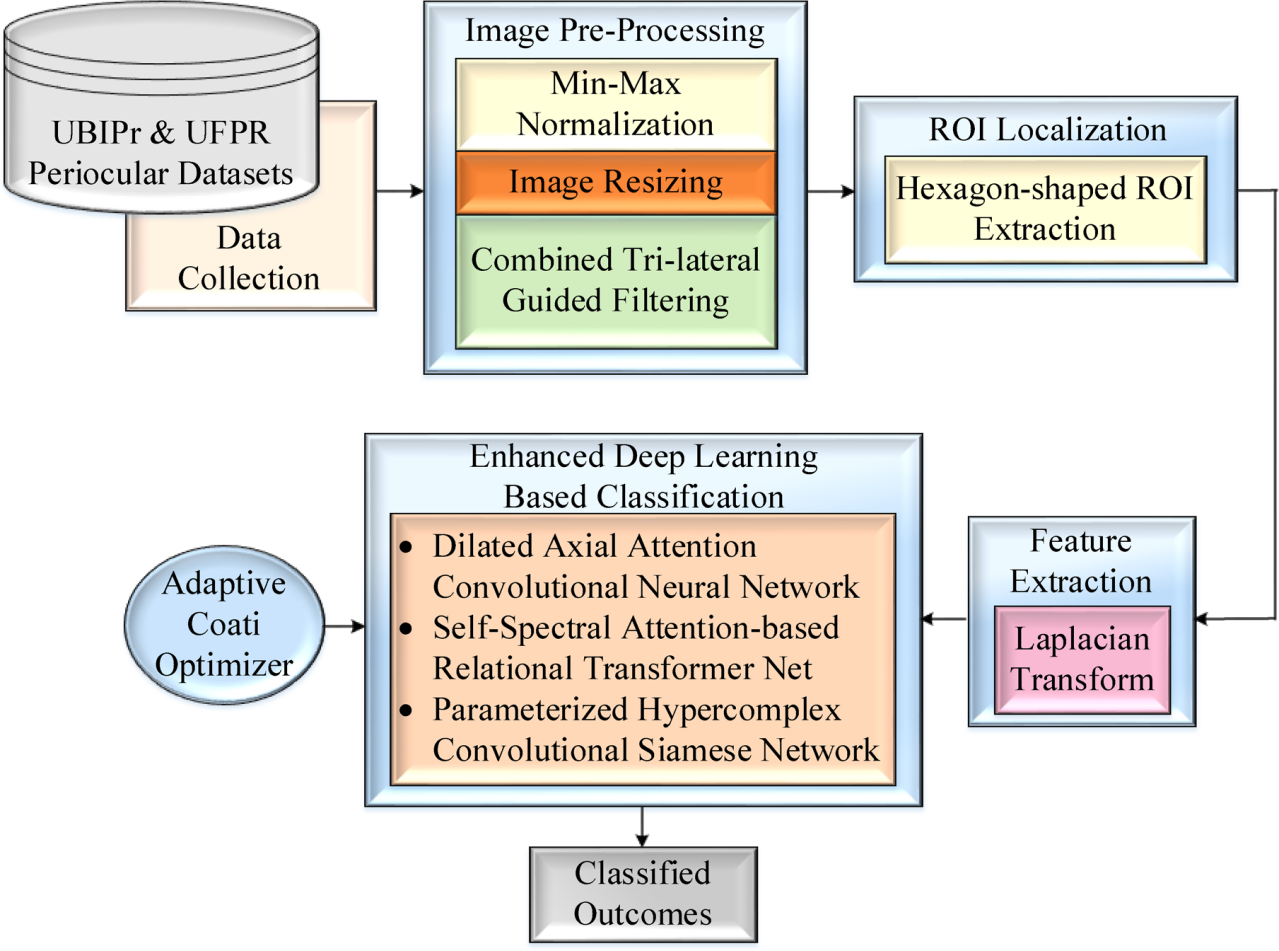
Block schematic of the envisaged EDLC framework.

Within this section, we discuss the intended EDLC framework, which is presented in Fig. 1. To begin with, the input images emerge from the dataset and are subsequently pre-processed using image resizing, normalization, and CTri-LGF. The ROI localization is performed based on a hexagon shape. Next, the Laplacian transformation is applied for the extraction of features, and subsequently, the categorization is done with three enhanced deep-learning classifiers such as DAA-CNN, SSA-RTNet, and PHCSN. Furthermore, the hyper-parameters of these classification frameworks are altered using the ACoOA.

### Pre-processing

Preprocessing is a crucial stage that can be performed prior to feature extraction^38^. Image pre-processing procedures include image resizing, filtering, and other operations for improving the image quality. The proposed method employs image resizing, normalization, and CTri-LGF in the pre-processing module.

#### Image resizing

If the original image size is less than the model requirement of input (224 × 224), center cropping^39^ will fill the image’s surrounding region based on zero padding to match the criteria. On the other hand, if the ordinary image size exceeds the model input condition, the center crop can snip the periocular image from the center to achieve the target size.

#### Min–max normalization

The image normalization process generally changes the distribution and range of pixel values to embellish the stability, accuracy, and convergence speed of the model^40^. In the EDLC framework, min–max normalization is applied for normalizing the image. According to Eq. (1), the min–max normalization approach processes every pixel intensity.

1 $$y_{Norm} = \frac{{y_{j} - Mini\left( y \right)}}{Maxi\left( y \right) - Mini\left( y \right)}$$

where *y*_*Norm*_ specifies the normalized pixel intensity, *y*_*j*_ resembles the image’s pixel intensity value, *Maxi(y)* and *Mini(y)* depict the maximum and minimum pel intensity value of the image.

#### Combined tri-lateral guided filtering

In the field of visual processing, edge-preserving filters are seen as ongoing research in recent times. Edge-preserving smoothing filters like bilateral filters, guided filters, and weighted least squares prevent edge distortion or blurring in an image. To embellish the performance of denoising in images, the proposed technique uses CTri-LGF.

In CTri-LGF, two images; an input image and a guided image are considered as input. Later, the guided image is divided into separate color channels, (for instance, Red, Green, and Blue)^41^, since the proposed method considers the guided image as a color image. A mean filter is applied to the three channels, and the cumulative sum of the x and y axes is taken to improve the pixel values. For mean computation, a box filter^42^ is utilized. The output image *P* of the box filter is normalized as follows:

2 $$P\left( {X,Y} \right) = \frac{1}{{\left( {2s + 1} \right)^{2} }}C\left( {X,Y} \right)$$

where, $P\left( {X,Y} \right)$ specifies the area of the kernel (square window), *S* indicates the kernel radius, and $C\left( {X,Y} \right)$ resembles the cumulative sum of intensity values within the kernel centered at pixel $\left( {X,Y} \right)$.

The local mean and covariance values are utilized to create a local covariance matrix in each color channel for each pixel. Next, the smoothing filter coefficients are calculated by employing the local covariance matrix. By means of the calculated filter coefficients, the guided filter is applied separately to each color channel of the input image. To do this, the neighborhood’s pixel values are weighted and averaged; the weights are established by the filter coefficients. The effectiveness of guided filtering is based on the tunable regularization parameter. After each color channel has been subjected to the guided filter, the filtered color channels are combined to create the final filtered color image.

### Hexagon-shaped ROI localization

In face recognition, the most prevailing ROI extraction techniques have utilized set-size rectangular ROI depending on a few reference points, such as the center of the eye or the center of the iris, disregarding the shape of a person’s periocular region. However, if the ROI detection technique incorrectly identifies a region, it can significantly impact subsequent stages of the pipeline. For instance, the extracted features may lack crucial information for accurate classification when the ROI cannot capture the periocular region accurately. The classifier depends on the quality of input features. Misclassified ROIs can create irrelevant information, tending to maximized error rates, minimized accuracy, and potential overfitting in the model. The errors in ROI detection can propagate through the pipeline, compounding inaccuracies and minimizing the reliability of the system for person identification and gender classification. Research on facial and fingerprint recognition systems^43^ underscores that inaccuracies in ROI localization can tend to increase false rejection and acceptance rate. The need for hexagon-shaped ROI extraction algorithms in periocular biometrics has been applied in the proposed method owing to the inability of triangular or rectangular ROIs to extract detailed structural features. A closed geometric figure with straight sides with all its angles equal in measure and all its sides equal in length is a polygon. Due to the simplicity of a regular polygon, the proposed model presents a hexagon-shaped ROI localization for extracting the periocular ROIs by considering the properties of a regular hexagon. The uniformity and symmetry of a regular hexagon define the properties of a regular hexagon. In image processing applications, the usage of hexagons can result in a symmetrical and uniform region for image localization.

A regular hexagon’s center is regarded as its typical focal point. One technique to describe the hexagonal region while localizing a periocular image within a hexagon is through the consideration of the center and computing the lengths between the vertices and the center. This center-point approach is often employed in applications where symmetry and balance are crucial for precise localization. The extent of the localized region has been quantified by means of the hexagon area. Within the defined region, the hexagon’s balanced design permits an even distribution of information.

### Feature extraction using Laplacian transformation

The proposed model employs the Laplacian transformation to extract the features from the ROI regions^41^. When compared to existing techniques for feature extraction, the Laplacian transform has a number of benefits, especially in image processing and CV applications.

The proposed EDLC framework utilizes the Laplacian transformation for extracting features from the localized images. For person identification and GC, it is necessary to extract desirable and significant attributes like the outer boundary. In the first step, the Laplacian transformation has been utilized mainly to scale and locate edges and disruptions. In the second step, the bank of directional filters is utilized to establish irregular associations and establish linear structures. The Laplacian transformation^45^ employs a pyramidal low pass filter to obtain the low-frequency components out of the periocular image, which are then removed from the original depiction, setting out only the differential periocular image with high-frequency features. Further, the technique is repeated several times using a factor (2, 2) for sampling. The significant features extracted using the Laplacian transformation include edge features such as texture, shape, edge, and structural characteristics. The statistical features computed using the Laplacian transform encompass mean, kurtosis, skewness, and variance of the pixel intensities. Additionally, it extracts high-frequency components like Fourier coefficients or wavelet features and dominant features, respectively. The distinctive colors and shapes are emphasized by the structural characteristics of localized images obtained using edge-based features.

In the proposed framework, three different deep learning classifiers, DAA-CNN, SSA-RTNet, and PHCSN, are employed for classifying the periocular images.

### Enhanced deep learning classifiers

#### Dilated axial attention convolutional neural network (DAA-CNN)

In general, CNNs represent an enhanced category of neural networks that can be used to implement diverse mathematical learning approaches such as gradient descent, backpropagation (BP), and regularization. CNN principles are based on three major essential layers: the convolutional layer, the pooling layer, as well as the fully connected (FC) layer^46,47^. One disadvantage of CNN is the usage of gradient descent to reduce the variability between the desired value and the achieved response from the network. Indeed, while applying gradient descent, the solution can fail to provide the best possible global solution and instead become stuck in the local minimum^48^. Thereby, a prevailing model named DAA-CNN^49,50^ is introduced with specific enhancements and modifications to meet the task of person identification and gender classification. The ability to effectively capture local and global contextual information using axial attention processes and dilated convolutional layers is beneficial. The DAA-CNN can extract hierarchical information at many scales by extending the receptive field of neurons without appreciably increasing computational complexity by adding dilated convolutions. It also efficiently captures the spatial relationships as well as the fine-detailed features.

**Fig. 2:**
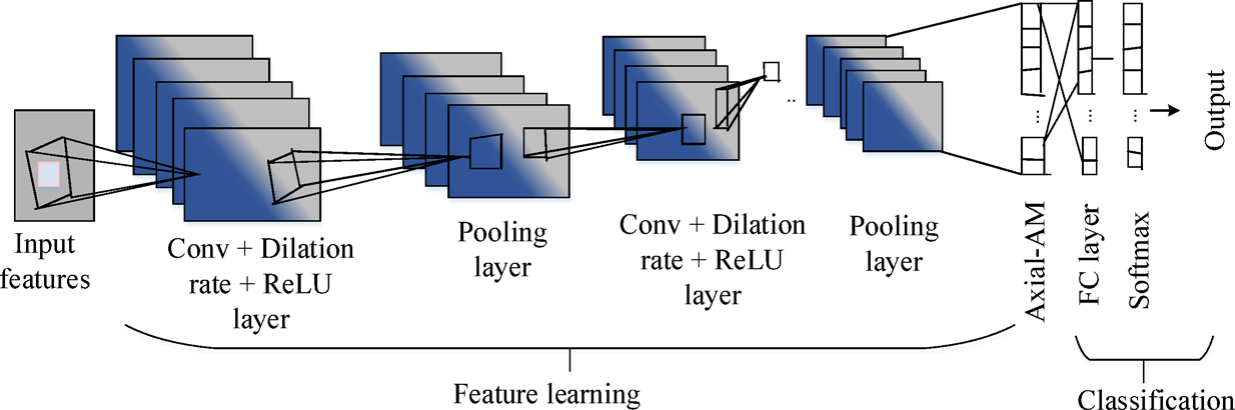
Architecture of dilated axial-attention convolutional neural network model.

In the proposed EDLC model, DAA-CNN comprises different layers, particularly the dilated convolution layer, pooling layer, axial attention layer, fully connected (FC) layer, and softmax layer. The convolutional layers, which comprise distinct feature vectors, are a crucial component of DAA-CNN. The convolution layer has a high number of spatial dimensions that are sub-sampled by the pooling layer to achieve the corresponding output. The architecture of DAA-CNN is presented in Fig. 2.

The DAA-CNN can extract the original image’s regional features. The principal intent of the learning process is to optimize the kernel matrices for making better significant features. ReLU is employed as the activation function for the neurons through a function g(y) = Max(y,0). For minimizing neural network error, cross-entropy (CE) loss^47^ is considered as fitness/loss function. The loss function can be indicated as mentioned in Eq. (3).

3 $$L = \sum_{k = 1}^{P} \sum_{j = 1}^{N} - D_{k}^{\left( j \right)} \log Z_{k}^{\left( j \right)}$$

where P indicates the number of samples, $D_{k}$ specifies the envisioned output vector, and $Z_{k}$ implies the achieved output vector of the nth class, which can be attained with the help of Eq. (4).

4 $$Z_{k}^{\left( j \right)} = \frac{{e^{{g_{k} }} }}{{\mathop \sum \nolimits_{j = 1}^{N} e^{{g_{j} }} }}$$

The weight penalty is used to develop the function L to add an α value for enhancing the weight value as shown in Eq. (5).

5 $$L = \sum_{k = 1}^{P} \sum_{j = 1}^{N} \left( { - 1} \right)*D_{k}^{\left( j \right)} \log Z_{k}^{\left( j \right)} + \frac{1}{2}\alpha \sum_{J} \sum_{L} \omega_{j,l}^{2}$$

where, $\omega_{j}$ resembles the connection weight, j is the layer of m connections, and L specifies the overall number of layers.

Further, the axial-attention scheme^51^ has been levied to boost the outcomes of CNN as an augmented unit by fragmenting the common self-attention. Here, width-axial attention has been considered, and supposed that pixels with position $\left( {H,k} \right)$ in a feature map contains the vector. Also, the self-attention formulation has been provided in Eq. (6):

6 $$z_{jk} = \sum_{h = 1}^{{H_{ei} }} \sum_{w = 1}^{{W_{id} }} Softmax\left( { r_{jk}^{T} l_{hw} } \right)V_{hw}$$

where, $z_{jk} \varepsilon R^{{C_{out} *H_{ei} *W_{id} }}$ resembles the outcome of the self-attention layer. Thereby, the width-axial attention has been described in Eq. (7):

7 $$z_{jk} = \sum_{w = 1}^{{W_{id} }} Softmax\left( { r_{jk}^{T} l_{jw} + r_{jk}^{T} s_{jw}^{r} + l_{jw}^{T} s_{jw}^{l} } \right)\left( {V_{jw} + s_{jw}^{V} } \right)$$

where, $s^{r} ,s^{l} and s^{V}$ location term included into the vector of $r,l and V$ while traversing point by point along the width direction, considerably. In the same way, the height-axial attention is described in Eq. (8):

8 $$z_{jk} = \sum_{h = 1}^{{H_{ei} }} Softmax\left( { r_{jk}^{T} l_{jh} + r_{jk}^{T} s_{jh}^{r} + l_{jh}^{T} s_{jh}^{l} } \right)\left( {V_{jh} + s_{jh}^{V} } \right)$$

A dilated convolution unit^52^ is utilized to capture more information from the global interactions. By concatenating the entire information together, the final outcomes are attained. The dilation operation is specified in below Eq. (9):

9 $$Z\left( {X,Y} \right) = \varepsilon \left\{ {\mathop \sum \limits_{jk} g\left( {t + j \times S, Y + k \times S} \right) \times h\left( {j,k} \right) + \mu } \right\}$$

where $\varepsilon$ represents the activate function, $\mu$ specifies the biased unit, $s$ denotes the changeable parameter. In this case, ACoOA is employed to achieve the lowest possible error value. As a result, the classification performance can be enhanced for detecting the periocular face images.

#### Self-spectral attention-based relational transformer Net (SSA-RTNet)

**Fig. 3:**
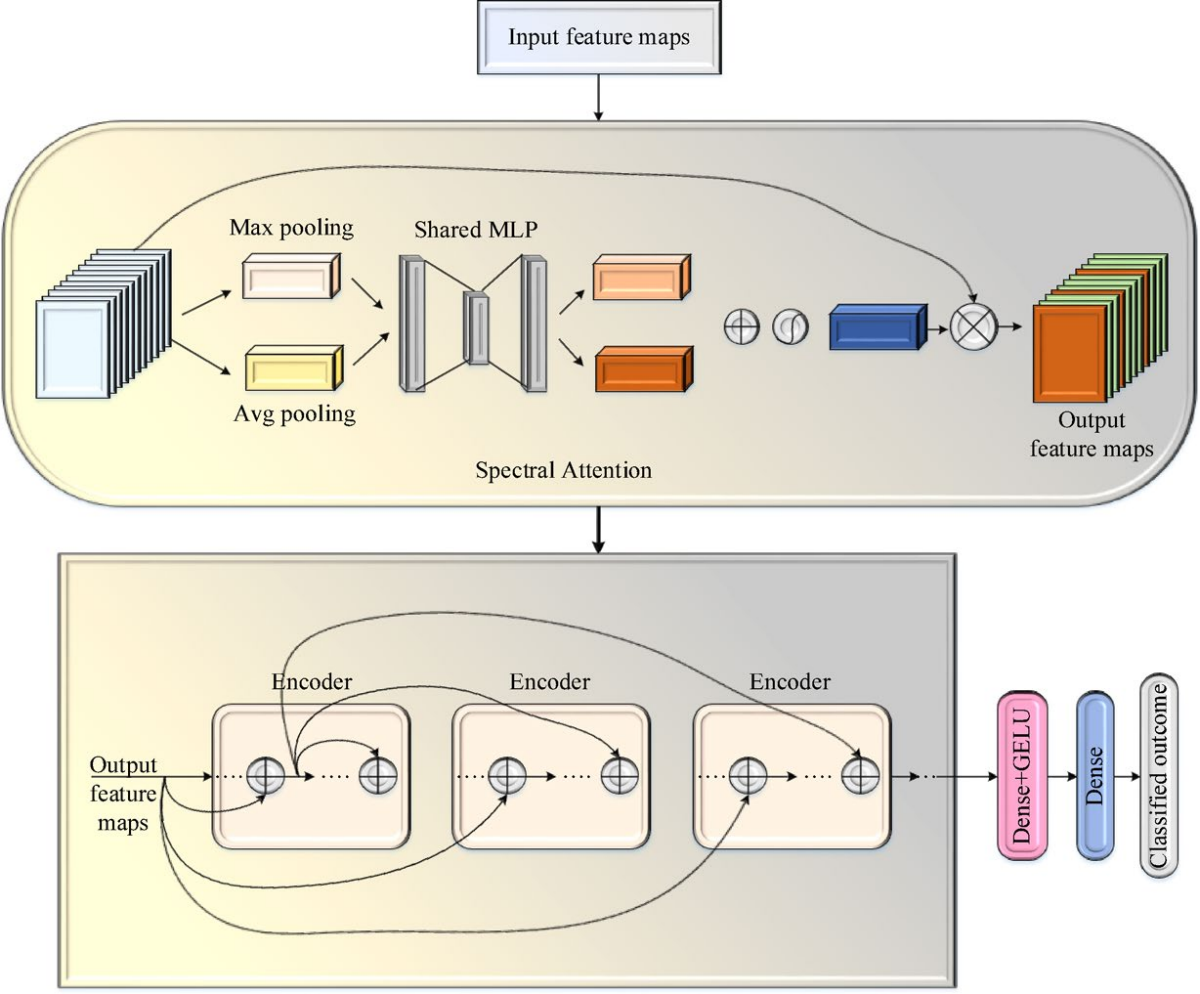
Architecture of SSA-RTNet.

The architecture of the SSA-RTNet is demonstrated in Fig. 3, for periocular face image detection, based on spectral attention.

The SSA-RTNet model first converts the input data into several sequences using the spectral attention (SA) module and subsequently employs their sequence of linear embeddings as transformer model input^53^. A multi-head self-attention (MHSA) module with a residual structure is used to learn the feature information^54^.

##### Spectral attention block

In the SSA-RTNet model, the SA module is applied to bolster the feature learning potential of the neural network. The intent of utilizing SA is to gather the features relevant to periocular face image classification by varying the spectral information weight. Global maximum coupled with global average pooling are used to learn the feature information. Two distinct pooling techniques extract more abstract features, followed by activation functions and two FC layers, to produce two-pooling channel information. Further, the two feature channel weights are combined by employing the correlation technique. Finally, the weight of the input feature map has been modified by multiplying the latterly used feature weight per input feature mapping in order to extract higher-level feature information.

##### Multi-head self-attention

The self-attention module enhances the attention module, which mitigates the reliance on extraneous data and permits for superior capturing of internal data correlation or representative information. In the EDLC framework, the self-attention variant, particularly the multi-head self-attention mechanism, is used to learn image attributes. The SSA-RTNet pipeline comprises of a MHSA module with several self-attention progression techniques. MHSA initially remap $Y_{j}$ to $r_{j} ,l_{j} ,w_{j}$ by using three initialization transformation matrices $X_{r} , X_{l}$ and $X_{w}$ as shown in Eq. (10)-(12):

10 $$r_{j} = X_{r} Y_{j}$$

11 $$l_{j} = X_{l} Y_{j}$$

12 $$w_{j} = X_{w} Y_{j}$$

where, $Y_{j}$ denotes the input features processed initially, followed by the detection block. The resultant flat 2D block of a similar size $X_{r} ,X_{l}$ and $X_{w}$ resemble three dissimilar weight matrices that linearly vary the original vector of input and accomplish three dissimilar linear transformations on each input for obtaining the intermediate vectors $r_{j} ,l_{j}$ and $w_{j}$, thereby maximizing the diversity of the model feature sampling.

Then, the weight vector $\hat{b}_{k}^{m}$ calculated according to $r_{k}$ and $l_{n}$ parameters, and it is described in Eq. (13):

13 $$\hat{b}_{k}^{m} = \frac{{{\text{exp}}\left( {\frac{{r_{k} .l_{n} }}{\sqrt e }} \right)}}{{\mathop \sum \nolimits_{P + 1} {\text{exp}}\left( {\frac{{r_{k} .l_{P + 1} }}{\sqrt e }} \right)}}$$

where $P$ indicates the number of flattened 2D blocks. Then, the dot product operation is applied on $r_{k}$ and $l_{n}$, and divided by $\sqrt e$, where $e$ implies the dimensions of $r$ and $l$ for normalizing the data considerably. Subsequently, the weight vector $\hat{b}$ defines the outcome by means of the softmax function. The $\hat{b}$ vector depends on all $l$ vectors and the $q$ vector, and so the above equation constructs in total $P + 1$ vectors with the length of $P + 1$ per vector.

Next, a weighted average operation is performed to compute vector $d_{j}$ as shown in Eq. (14):

14 $$d_{j} = \mathop \sum \limits_{j} \hat{b}_{k}^{n} w_{n}$$

The output vector of the above equation is considered the weighted average of every $w$ vector, with weights offered by $\hat{b}$ vector.

##### Encoder block

**Fig. 4:**
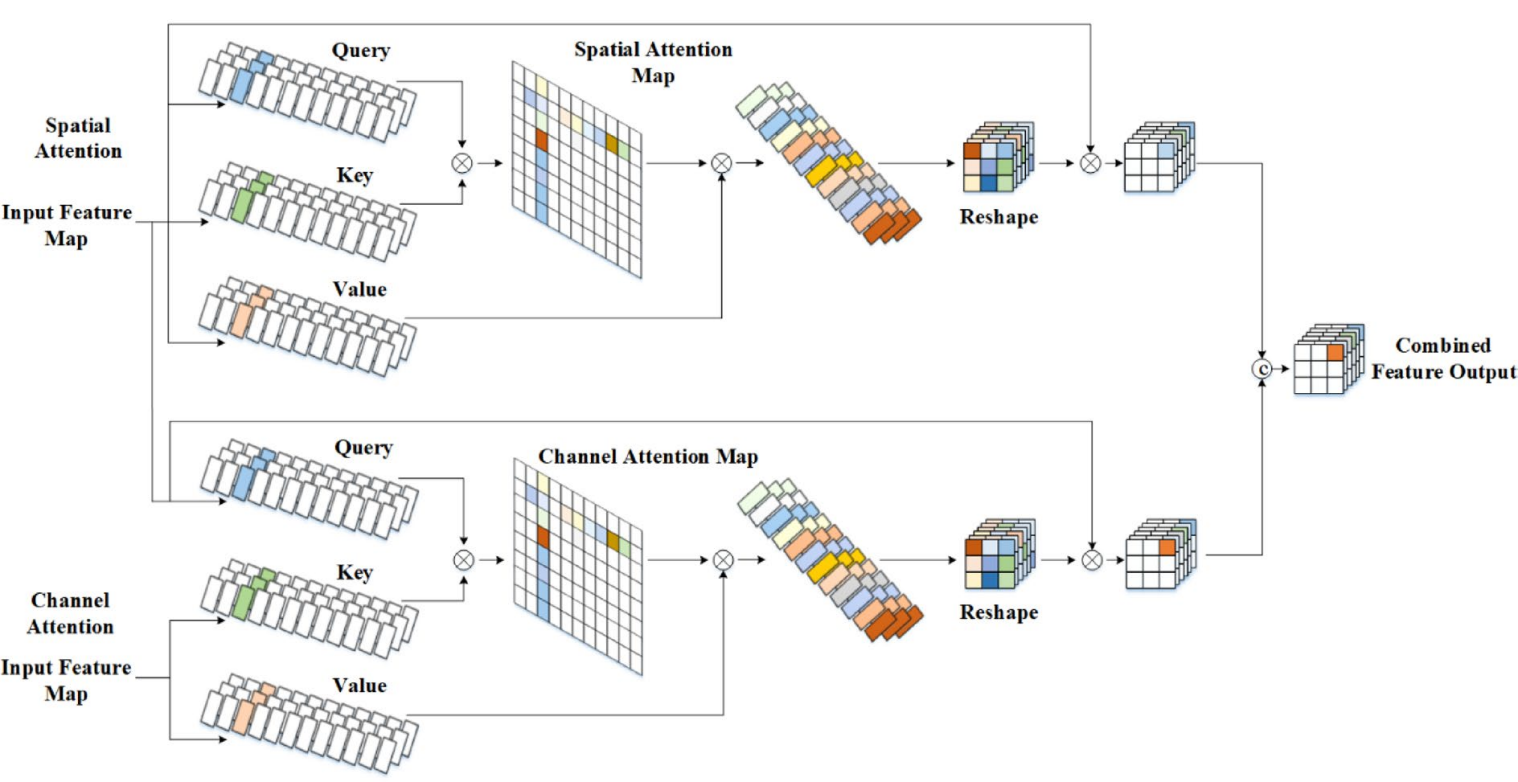
Architecture of encoder block of SSA-RTNet.

The encoder part is designed using a relational transformer block (ReTB)^55,56^. In order to capture dependencies between persons and their gender information, the ReTB is composed of a self-attention as well as a cross-attention head. The query, key, and value generators in each head are $g_{j} ,k_{j} ,v_{j} ;j \in \left\{ {S,C} \right\}$. The trainable linear embeddings are effected using a $3 \times 3$ convolution and a reshaping procedure. Figure 4 depicts the architecture of the encoder block of SSA-RTNet.

Equation (15) and Eq. (16) provide the description of the pairwise query as well as crucial computations in the cross-attention head as well as the self-attention head.:

15 $$F_{C} \left( {f_{m} ,f_{w} } \right) = K_{C} \left( {f_{w} } \right)^{T} q_{C} \left( {f_{m} } \right)$$

16 $$F_{S} \left( {f_{m} } \right) = K_{C} \left( {f_{m} } \right)^{T} q_{S} \left( {f_{m} } \right)$$

where, the cross-attention and self-attention heads are indicated by C and S, correspondingly. It is pivotal to highlight that the key is derived entirely from input periocular features $f_{m}$, in contrast to the self-attention head that generates the query from the feature. The cross-attention head integrates the context information by generating the key from the feature $f_{w}$.

The two heads, distinct attentive traits are then computed in the manner described below:

17 $$g_{C} \left( {f_{m} ,f_{w} } \right) = V_{C} \left( {f_{w} } \right)^{ } softmax\left( {F_{C} \left( {f_{m} ,f_{w} } \right)} \right)$$

18 $$g_{S} \left( {f_{m} } \right) = V_{S} \left( {f_{m} } \right)^{ } softmax\left( {F_{S} \left( {f_{m} } \right)} \right)$$

Residual learning is applied to every head, and the results are as follows:

19 $$f_{j} = w_{j} g_{j} \left( {f_{m} ,f_{w} } \right) \oplus f_{m} ;j \in \left\{ {S,C} \right\}$$

where, $\oplus$ operation is carried out by a residual interaction of element-wise addition, $w_{j}$ indicates the implementation of linear embedding towards $1 \times 1$ convolution. Due to this, the self-attention head accurately captures long-range relationships, by computing the response at a position, as a weighted sum of the features in all segments. The purpose of the head is to further improve the boundaries of enormous patterns and to differentiate persons. The cross-attention head uses the contextual features to query. The final output is obtained by concatenating the attributes $f_{s}$ from the self-attention as well as $f_{c}$ from the cross-attention head:

20 $$f_{out} = \left[ {f_{s} ;f_{c} } \right]$$

where $\left[ {.;.} \right]$ resembles concatenation.

#### Parameterized hypercomplex convolutional siamese network (PHCSN)

In this section, the PHCSN is described for periocular image classification. PHCSN combines the advantages of parameterized hypercomplex convolutional (PHC) and siamese network to improve the accuracy rate. The convolutional siamese network (CSN) generally has the ability to efficiently acquire discriminative features from the input for providing person identification and gender classification. CSN is trained to learn robust and discriminative representations, such as gender-related attributes and periocular features. The shared convolutional layers of Siamese architecture make it simpler for the network to acquire features that are impervious to changes in lighting and posture. Additionally, the network can learn nuanced features that are indicative of individual identities and gender qualities by aiming at subtle changes through the pairwise evaluation tactic included in CSN. Moreover, this method improves the capability of a network to generalize across various persons.

**Fig. 5:**
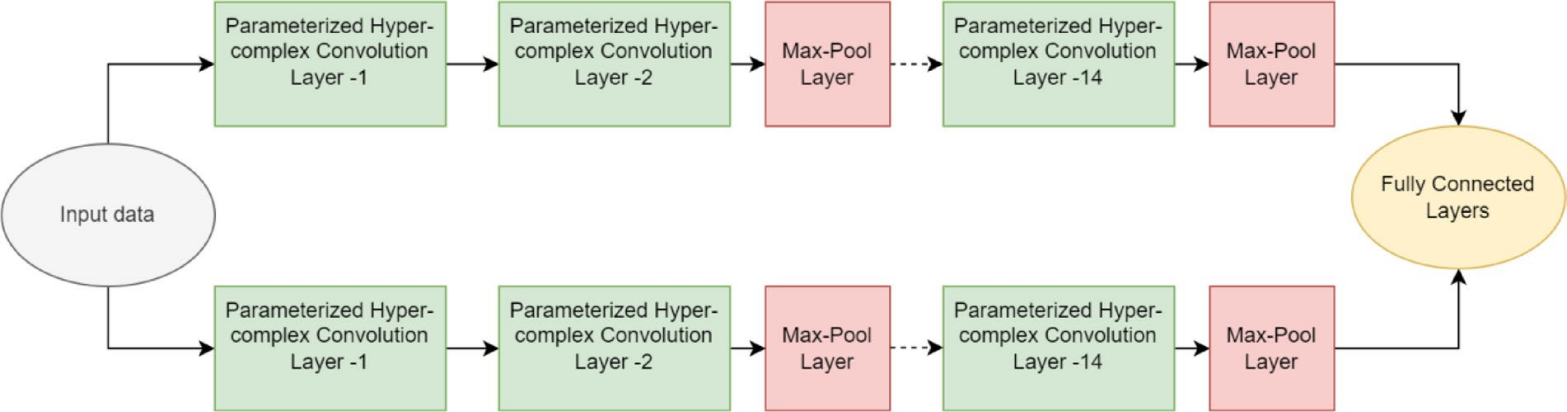
Architecture of parameterized hypercomplex convolutional siamese network.

In the proposed EDLC model, CSN is considered a class of CNN-based model that generally comprises two similar CNNs. The dual CNN exhibits an identical configuration^57^ with similar shared weights and parameters. The sub-networks are combined by the loss function and determine the similarity metrics that compute the Euclidean distance among the learned feature vectors. In CSN, y_1_ and y_2_ are considered as the inputs and x indicates the shared parameter vector, which is tuned in the training stage. The architecture of CSN is shown in Fig. 5.

$G_{x} \left( {y_{1} } \right) and G_{x} \left( {y_{2} } \right)$ defines the learned feature vectors through each of the CNNs that the Siamese network encompasses. The outcome of the CSN determines the similarity between feature vectors, and it is described below in Eq. (21).

21 $$E_{x} = G_{x} \left( {y_{1} } \right) - G_{x} \left( {y_{2} } \right)$$

Moreover, the hypothesis in the proposed model is that the features of the same periocular face image can have the same feature vectors, thereby the distance is zero. The features of the different periocular face images can have dissimilar feature vectors, so their distance is higher. In the suggested model, CSN has employed Euclidean distance since it is widely applied in various applications and has achieved better performance. The measure of the learned similarity function is given in Eq. (22).

22 $$E_{x} \left( {y_{1} ,y_{2} } \right) = G_{x} \left( {y_{1} } \right) - G_{x} \left( {y_{2} } \right)_{2}$$

Moreover, the CSN utilized a contrastive loss function over the training stage, and it is specified below as follows:

23 $$Loss\left( {x,z,y_{1} ,y_{2} } \right) = \frac{z}{2} E_{x} \left( {y_{1} ,y_{2} } \right)^{2} + \frac{1 - z}{2}\left( {Maxi\left\{ {0,n - W_{x} \left( {y_{1} ,y_{2} } \right)} \right\}} \right)^{2}$$

where, $n > 0$ states the constant termed as margin and y resembles the binary label allocated to input pairs $y_{1}$ and $y_{2}$, thereby $z = 0$ if the input is not a specific class of periocular face images, and else $z = 1$.

Besides, it is noted that the periocular face images belong to the identical person ($z = 1$) their distance contributes to the loss function. On the other hand, when they belong to dissimilar individual ($z = 0$), their distance equal to or less than $n$ contribute. Therefore, reducing $Loss\left( {x,z,y_{1} , y_{2} } \right)$ with respect to χ can outcome in a smaller value of $E_{x} \left( {y_{1} , y_{2} } \right)$ for the same individual and a larger value of $E_{x} \left( {y_{1} , y_{2} } \right)$ for different individuals. Here, the ACoOA is engaged to minimize the loss by selecting the optimal parameters.

#### Improvement of CSN with parameterized hypercomplex convolution layer

The PHC layer is a method that uses hypercomplex numbers to expand the traditional convolution^58,59^. The weight tensor $I$, which employs the sum of Kronecker products to capture and arrange the convolution’s filters, serves as the foundation for the PHC layer. The suggested approach can be delineated as shown in Eq. (24).

24 $$\hat{Z} = PHC\left( {\hat{y}} \right) = I * \hat{y} + \hat{c}$$

where, $I \in R^{v \times g \times n \times n}$ is built using the sum of the Kronecker products between two learnable groups of matrices. Here, $n$ indicates the filter size, $g$ resembles the output dimensionality, and $v$ describes the input dimensionality of the layer. In more specific terms,

25 $$I = \mathop \sum \limits_{l = 1}^{r} \hat{B}_{l} \otimes \hat{G}_{l}$$

where, $\hat{B}_{k} \in R^{r \times r}$ describes the matrices that characterize the algebra rules with $l = 1,2, \ldots r$ , and $\hat{G}_{l} \in R^{{{ }\frac{v}{r} \times \frac{g}{r} \times n \times n}}$ indicates the $l^{th}$ batch of filters that are positioned rendering based on the algebra rules to produce the final weight matrix. It should be noted that $\frac{v}{r} \times \frac{g}{r} \times n \times n$ is employed to squared kernels, for 1D kernels, $\frac{v}{r} \times \frac{g}{r} \times n$ should be deliberated. The Kronecker product, a generalization of the vector outer product parameterized by $r$, is the fundamental element of this approach. The user can configure the hyperparameter $r$, to operate in a pre-defined real or hypercomplex domain, for instance, setting $r = 2$ facilitates the PHC layer in the complex domain, or setting $r = 4$ (quaternion) or alter it to acquire the optimum model performance. During training, the values of the matrices $\hat{B}_{l}$ and $\hat{G}_{l}$ are realized in order to build the final tensor $I$.

Less values for $n$ and a greater number of filters in layers $\left( {v,g = 256,512, \ldots } \right)$ are usually used in real-world applications. Consequently, $vgn^{2} \gg r^{3}$ is often true. Therefore, the degrees of freedom for the PHC weight matrix can be roughly estimated to $O\left( {vgn^{2} /r^{3} } \right)$. Compared to a typical convolutional layer, the PHC layer employs fewer parameters $1/r$, due to Kronecker products. Furthermore, PHC layers are superior to data with associated channels, such as color images, because of the weight sharing between several channels. This aids in capturing latent intra-channel relations that are missed by conventional convolutional networks because of the inflexible structure of the weights. The PHC layer can subsume hypercomplex convolution rules, and the hyperparameter $r$ expresses the necessary domain. Interestingly, setting $r = 1$ can also be used to describe a real-valued convolutional layer. Since typical real layers do not use parameter sharing, the entire set of filters is contained in $\hat{G}^{v \times g \times n \times n}$, and the algebraic rules are specified by $\hat{B} \in R^{1 \times 1}$.

### Parameter tuning based on adaptive coati optimizer

In the proposed model, hyper-parameter tuning is accomplished using the ACoOA to optimize the performance of periocular face image classification. The intricate procedure of learning hyper-parameters often results in training errors for the EDLC model. Traditional Coati Optimization Algorithm (CoOA)^60^ tends to suffer from early convergence and requires an increased number of iterations. To address this, ACoOA is used to optimize the parameters, reducing training errors and improving model performance. Chebyshev chaos mapping^61^ is done during the initialization phase of ACoOA, which minimizes the iteration count and competently prohibits early convergence. At the start of the ACoOA, the coatis’s location is randomly initialized, and it is designated as follows,

26 $$A_{j} :a_{j,k} = LoB_{k} + \Omega_{cm} u.\left( {UpB_{k} - LoB_{k} } \right), j = 1,2,3, \ldots R, k = 1,2,3, \ldots .p$$

where, $p$ states the number of decision variables, $\Omega_{cm}$ specifies the Chebyshev map, $a_{j}$ exemplifies the location of $j^{th}$ coati, $R$ designates the number of coatis, $a_{j,k}$ describes the value of $k^{th}$ decision variable, $UpB_{k}$ designates the upper bound, $u$ implies the arbitrary real number within the interval $\left[ {0,1} \right]$ as well as $LoB_{k}$ indicates the bottom limit of $k^{th}$ control variable.

Once initialized, each coati is assigned a fitness score based on a specific fitness function that aims to minimize the loss function.

27 $$Fit_{fun} \left( A \right) = Mini \left( { loss function } \right)$$

where, $Fit_{fun} \left( A \right)$ represents the fitness function. To further refine the hyper-parameters, ACoOA employs an exploitation strategy inspired by a predator escape mechanism. This strategy adjusts the coatis’ positions to improve their fitness scores, and if a new position results in improved fitness, it is accepted as the coati’s new location.

28 $$A_{j}^{S2} :a_{j,k}^{S2} = a_{j,k} + \left( {1 - 2u} \right).\left( {LoB_{k}^{LOCC} + t.\left( { U_{p} B_{k}^{LOCC} - LoB_{k}^{LOCC} } \right)} \right)$$

29 $$LoB_{k}^{LOCC} = \frac{{LoB_{k} }}{w}, U_{p} B_{k}^{LOCC} = \frac{{U_{p} B_{k} }}{w},{\text{ where}}\;w = 1,2,3, \ldots .W$$

where, $A_{j}^{S2}$ indicates the contemporary location deliberated for $j^{th}$ coati based on the exploitation of CoOA, $a_{j,k}^{s2}$ designates its $k^{th}$ dimension, $Fit_{{fun_{j} }}^{S2}$ delivers its fitness function value, $LoB_{k}^{LOCC}$ designates the local lower bound of $k^{th}$ decision variable, $w$ resembles the iteration counter, and $U_{p} B_{k}^{LOCC}$ recommends the local upper bound of $k^{th}$ decision variable. After the ACoOA run, the optimal solution acquired in each iteration is returned as the output.

## Results and discussion

In this section, the experimental analysis to analyze the results of the EDLC model for periocular image detection is provided. The proposed EDLC paradigm experimentation is carried out using the Python platform. The development environment is Keras 2.1.0; the GPU is GeForce GTX 1080. The operating system is Windows 10, the CPU is Intel Core i7-6700U, and the memory size is DDR3 8 GB. The dropout rate is fixed at 0.2, and the learning rate is given as 0.01. The EDLC model is optimized by exploiting the ACoOA with a batch size of 32 and using the loss function for reverse gradient propagation.

The data is partitioned during the experimentation to train and test the model. At every training round, 80% is accustomed to training, and the residual 20% is used for testing. Different evaluation metrics are exploited to analyze the EDLC model’’s functioning.

**Fig. 6:**
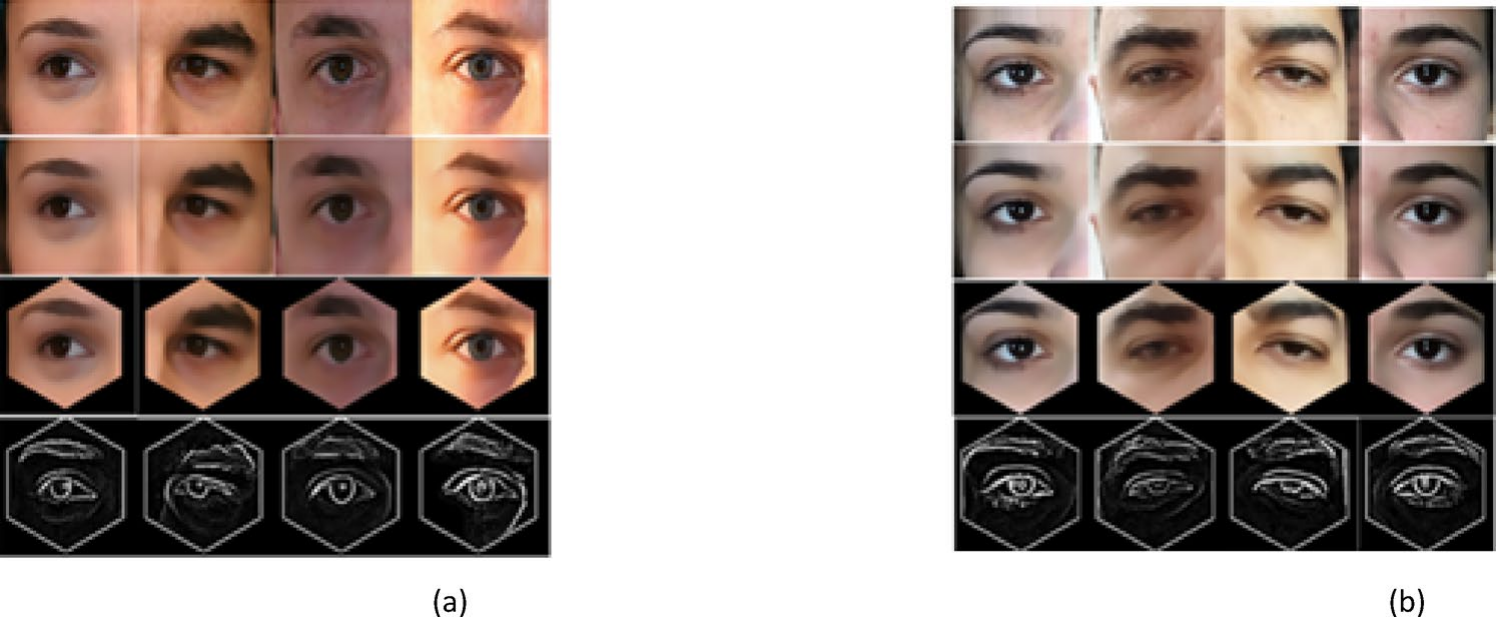
Simulation outcomes of the proposed method (**a**) UBIPr dataset (**b**) UFPR dataset. (Each image has 4 vertical partitions—Input image, preprocessed image, segmented image and feature extracted image).

In the following subsection, the particulars of the publicly available dataset employed in our experimentation are discussed initially, and then quantitatively, the simulated results are analyzed, and the EDLC model is compared against competitive models by computing the accuracy, precision, recall, and so on. The simulation findings of the proposed EDLC model are presented in Fig. 6.

### Benchmark datasets

For the experiment, the EDLC model considered two publicly accessible datasets, such as UBIPr and UFPR. The details of the dataset are provided below:

UBIPr dataset: The UBIPr dataset^62^ encompasses a total of 10,252 RGB periocular images in BMP configuration with an image resolution of 501 × 401 pixels from 344 subjects. Images in the UBIPr dataset are taken on the basis of 4 m to 8 m to encompass distance fluctuation. However, the acquisition is done in a controlled environment. Also, this dataset covers metadata documents for each image which incorporate focus of the iris coordinates of canthus points, midpoints, and end points of the brow in addition to information regarding gender, gaze angle, and pigmentation level. A portion of the images in the database experience occlusion complication due to eyeglasses or hair and pose variation in view of a tilted head.

UFPR-Periocular dataset: UFPR^63^ is considered a comprehensive color periocular dataset that was introduced in the year 2020. In general, it is designed to capture images in unrestricted conditions that include pragmatic noises attributable to blur and occlusion, as well as variations in angle, distance, and lighting. The dataset includes 16,830 pictures of both eyes from 1,122 participants. The image resolution ranges from 360 × 160 to 1862 × 1008 pixels according to the mobile apparatus, utilized to captivate the image. The UFPR periocular dataset includes biometric metadata such as age and gender.

### Performance indicators

To evaluate the execution of the EDLC framework, standard metrics such as accuracy, precision, recall, F1-score, and specificity are computed. The mathematical equation of these metrics is presented in Eq. (30–34). as follows:

30 $$Accuracy = \frac{{\Lambda_{tp} + \Lambda_{tn} }}{{\Lambda_{tp} + \Lambda_{tn} + \Lambda_{fp} + \Lambda_{fn} }}$$

31 $$Recall = \frac{{\Lambda_{tp} }}{{\Lambda_{tp} + \Lambda_{fn} }}$$

32 $$Precision = \frac{{\Lambda_{tp} }}{{\Lambda_{tp} + \Lambda_{fp} }}$$

33 $$F1 - score = \frac{2*Precision*Recall}{{Precision + Recall}}$$

34 $$Specificity = \frac{{\Lambda_{tn} }}{{\Lambda_{tn} + \Lambda_{fp} }}$$

where, $\Lambda_{tp}$ represents true positive, $\Lambda_{fp}$ suggests false positives, $\Lambda_{tn}$ signifies true negative, and $\Lambda_{fn}$ symbolizes false negatives.

### Performance evaluation

This section illustrates various experiments that are carried out to determine the efficacy and robustness of the periocular EDLC model from different aspects. The proposed EDLC model has introduced three different classifiers such as DAA-CNN, SSA-RTNet, and PHCSN.

For comparison, these three classifiers are contrasted against the competitor models such as Siamese neural network (SNN), stacked autoencoder (SAE), bidirectional long short-term neural network (BiLSTM), deep belief network (DBN), and CNN, respectively.

#### Quantitative analysis concerning various performance indicators

**Fig. 7:**
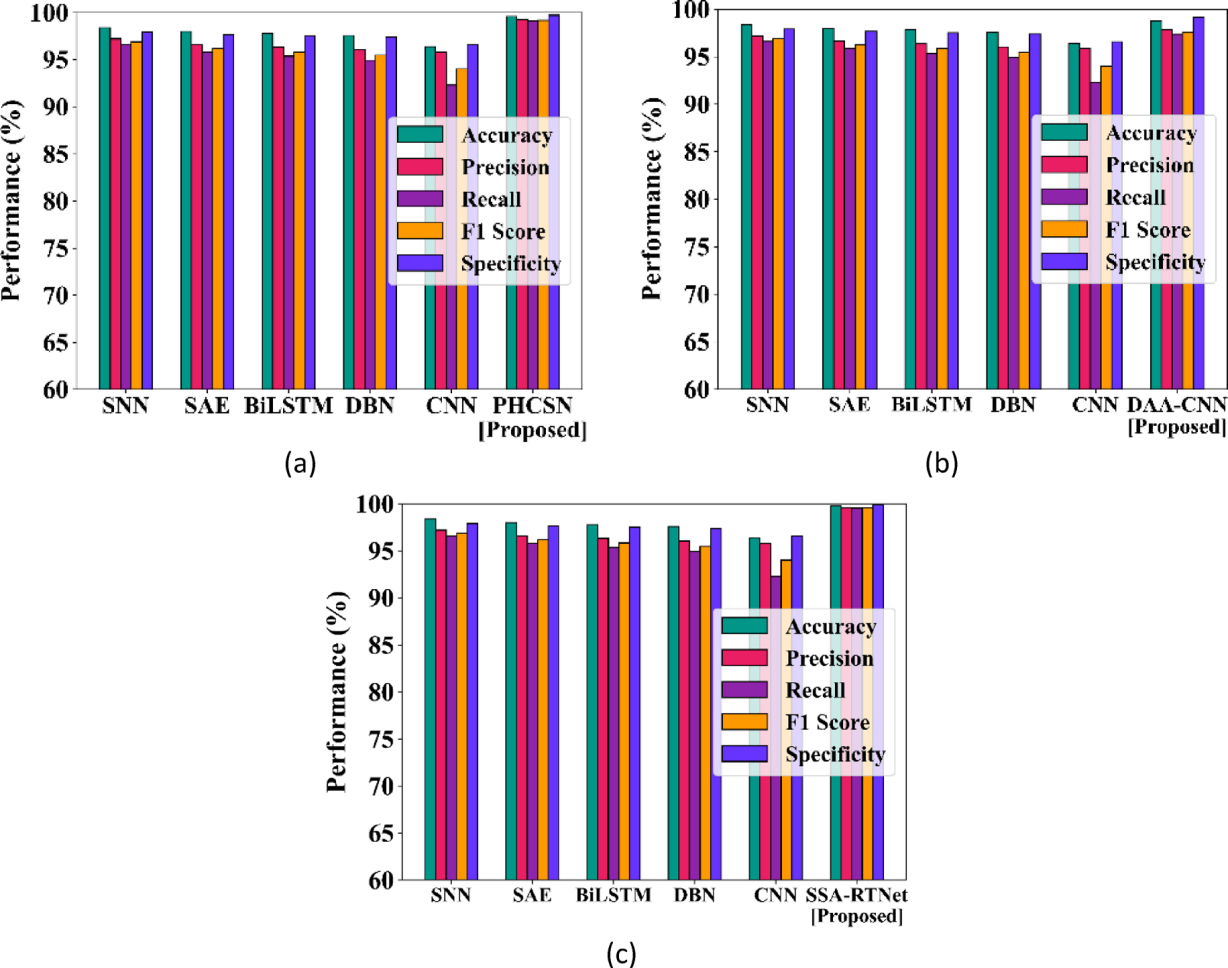
Analysis of EDLC model using UBIPr dataset (**a**) PHCSN vs competitor models (**b**) DAA-CNN vs competitor models (**c**) SSA-RTNet vs competitor models.

This portion of the sub-section presents the findings achieved by the EDLC model are analyzed regarding overall accuracy, precision, recall, F1-score, and specificity using UBIPr and UFPR datasets. The comparison of the EDLC model with competitive approaches using the UBIPr dataset is provided in Fig. 7.

**Fig. 8:**
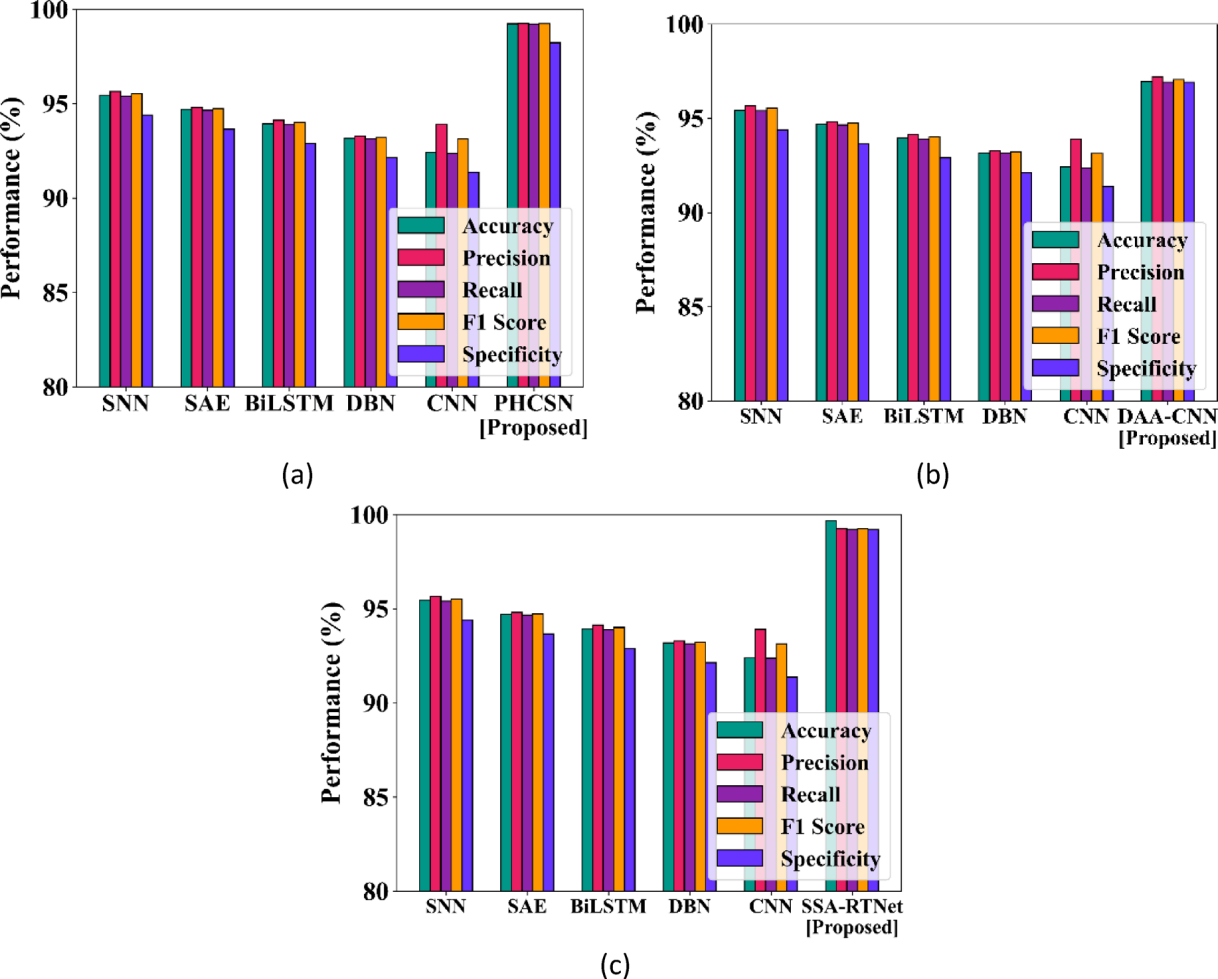
Analysis of EDLC model using UFPR dataset (**a**) PHCSN vs competitor models (**b**) DAA-CNN vs competitor models (**c**) SSA-RTNet vs competitor model.

In the same way, Fig. 8 demonstrates the comparison of performance with regard to different evaluation attributes by considering the UFPR-Periocular dataset. Considering the accuracy rate, the hybrid deep classifiers of EDLC models like DAA-CNN, SSA-RTNet, and PHCSN for periocular image classification have accomplished better performance than existing models, and it is clearly perceived in the graphical representations. It has been observed that the SSA-RTNet outperforms the other models with respect to the performance indicators.

The EDLC model offers an innovative solution by tackling the prevailing shortcomings of competitive models, such as overfitting problems, lack of proper weight updating, etc.

The maximum classification accuracy rates accomplished by the proposed SSA-RTNet, PHCSN, and DAA-CNN are 99.8%, 99.6%, and 99.20% for the UBIPr dataset and 99.67%, 99.24%, and 97.77% for UFPR-Periocular dataset. While perceiving the recall performance in Figs. 6 and 7, the graph evidently displays the efficiency of periocular image classification compared to competitive classifiers. Due to the adaptation of the ROI localization, Laplacian transform-based feature extraction, and the robust EDLC, the suggested model accomplished the best recall rate of the competitive architectures. Table 4 examines the EDLC model and competitive classifiers from the perspective of accuracy, F1 score, precision, recall, and specificity, with reference to UBIPr and UFPR datasets.

**Table 4:** Analysis of person identification for different performance metrics.

Dataset	UBIPr							
Metrics	SNN	SAE	Bi-LSTM	DBN	CNN	Proposed		
						SSA-TNet	PHCSN	DAA-CNN
Accuracy	98.41	98.01	97.80	97.60	96.40	99.8	99.24	99.20
Precision	97.22	96.62	96.33	96.05	95.81	99.61	99.24	98.05
Recall	96.59	95.82	95.36	94.92	92.29	99.56	99.12	97.36
F1_score	96.91	96.22	95.84	95.48	94.02	99.58	99.18	97.71
Specificity	97.92	97.65	97.52	97.38	96.58	99.86	99.73	98.19

Dataset	UFPR							
Metrics	SNN	SAE	Bi-LSTM	DBN	CNN	Proposed		
						SSA-TNet	PHCSN	DAA-CNN
Accuracy	95.45	94.70	93.94	93.18	92.42	99.67	99.24	97.77
Precision	95.65	94.81	94.13	93.29	93.11	99.26	99.26	97.38
Recall	95.41	94.66	93.89	93.14	92.37	99.23	99.23	96.92
F1_score	95.53	94.73	94.01	93.22	93.13	99.24	99.24	97.15
Specificity	94.40	93.66	92.89	92.14	91.37	99.23	98.23	95.92

Now, consider the precision and F1 score comparison with the EDLC model and competitive classifiers for periocular image detection and classification. In the graphical depiction, the precise experimental findings demonstrated that the EDLC model achieved improved precision in contrast to the competitive classifiers with reference to UBIPr and UFPR datasets.

Within the competitive classifiers, SNN has attained a precision close to our EDLC models, while DBN has achieved a very low precision rate. The quantitative evaluation of the F1 score showed that the EDLC model can recognize the class designations for the input periocular images. The visual representation shows that the outlined technique has accomplished a higher F1 score.

Correspondingly, the specificity performance also reached the most appealing outcomes over the two datasets, particularly on the UBIPr dataset, where it accomplishes the overall specificity of 99.86%, 99.73%, and 98.19% for SSA-RTNet, PHCSN, and DAA-CNN. The proposed EDLC model utilizes the improved algorithm to extract the image information on various scales. Thus, from the overall quantitative analysis, the EDLC model has obtained the most attractive outcomes. In contrast to competitive architectures, the performance of the EDLC model outperforms them for periocular image classification.

#### Analysis of accuracy and loss

**Fig. 9:**
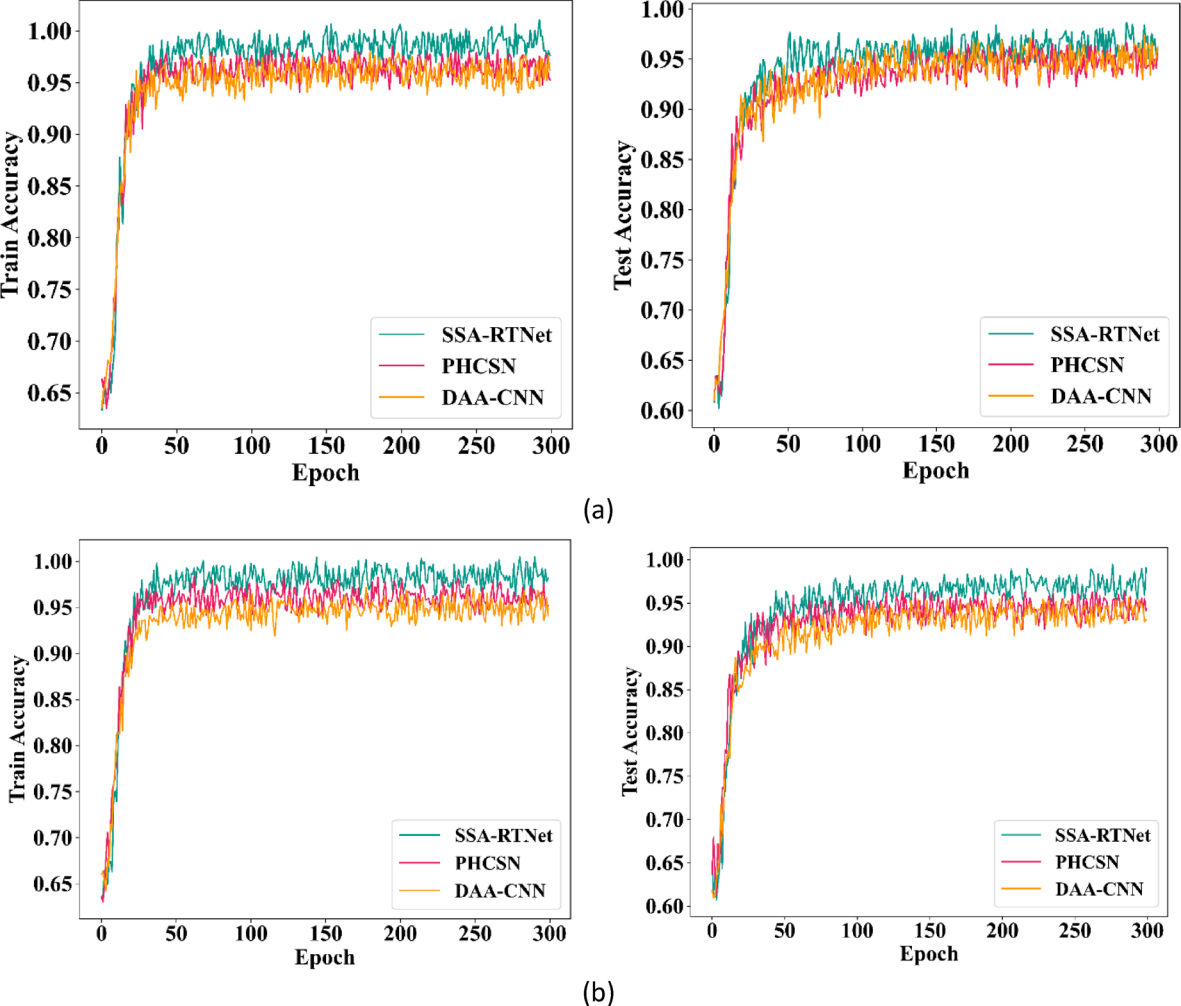
Training and testing accuracy (**a**) UBIPr dataset and (**b**) UFPR dataset.

In this sub-section, the accuracy and loss of EDLC are analyzed using training and test data for periocular image classification. The data is diverged to 80% for training along with the remaining 20% for testing. Figure 9 indicates the accuracy performance of the EDLC model during training as well as testing by employing UBIPr and UFPR datasets.

Here, the different classifiers of the EDLC model are analyzed. The outcomes show that as the count of training steps maximizes, the accuracy rate of each classifier, such as DAA-CNN, SSA-RTNet, and PHCSN, increases.

**Fig. 10:**
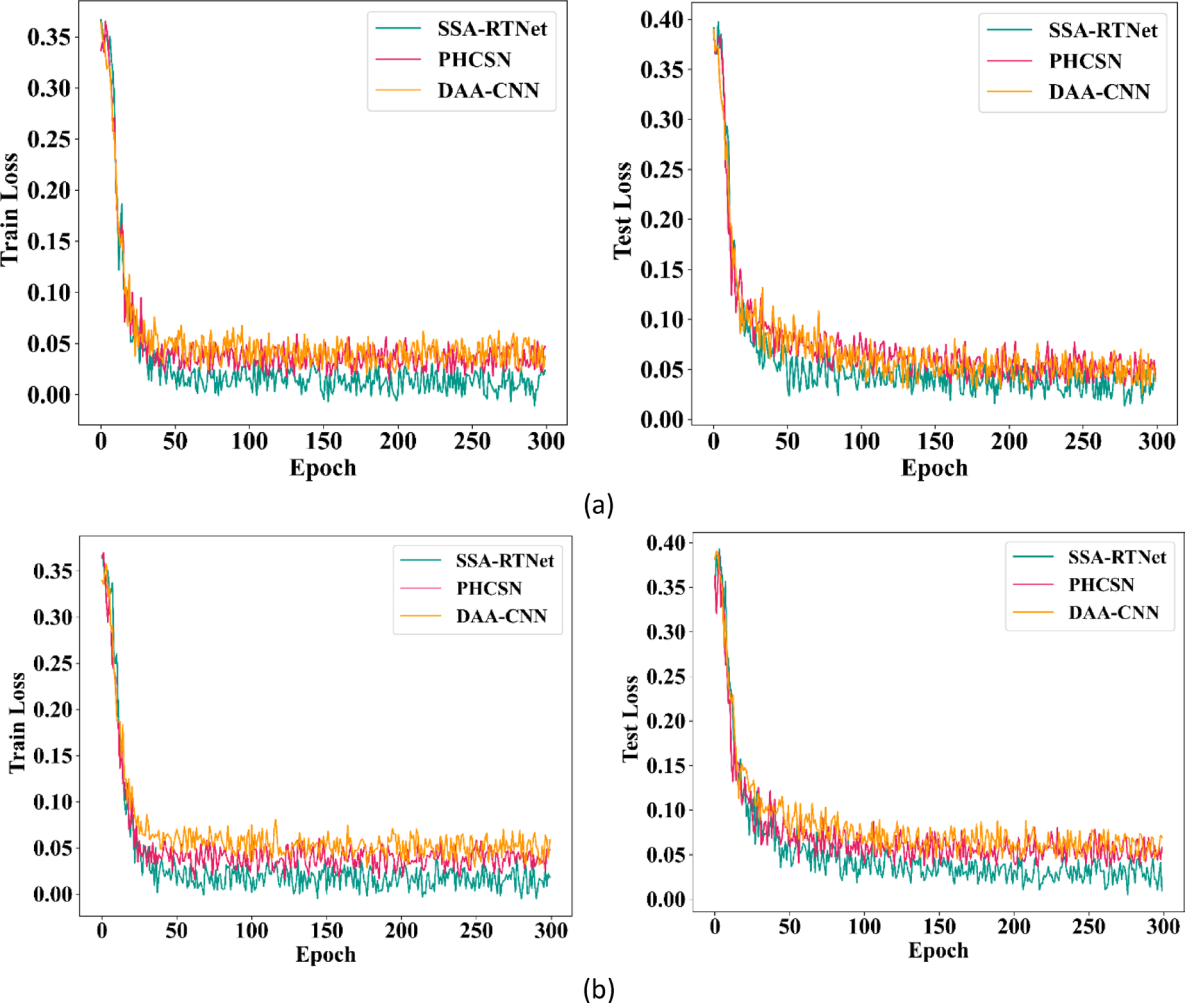
Training and testing loss (**a**) UBIPr dataset and (**b**) UFPR dataset.

Among the different classifiers of the EDLC model, SSA-RTNet has the best convergence speed. The proposed classifiers are trained for 300 epochs and have accomplished better accuracy. Moreover, the graph indicates that the intended classifiers converge better in 50 epochs. Figure 10 describes the loss of the EDLC model during training and testing by engaging UBIPr and UFPR datasets. From the graphical illustration, the DAA-CNN, SSA-RTNet, and PHCSN classifiers have obtained the most stable outcomes. The graphical illustration shows that, during the maximum number of training and testing epochs, the model is extremely stable. We can observe, that the loss percentage of DAA-CNN, SSA-RTNet, and PHCSN are extremely reduced, and as long as the the epoch count is increased, the resulting value is particularly poor. Therefore, it is evident, that the loss curve reveals that the EDLC model is quite consistent as the epoch count augments.

#### Benchmarking against current leading approaches

In this subsection, the implementation of the EDLC model is analyzed with recent approaches on unconstrained datasets such as UBIPr and UFPR with respect to accuracy.

The accuracy value is contrasted with state-of-the-art OCLBCP dual-stream CNN^24^, CNN + HOG^35^, Vision transformer-based model^36^, and LDA-CNN^64^. In addition, other recent methods, such as VGG19^65^ and Region-specific and subimage-based neighbor gradient models^66^ are also contrasted. The comparison of the proposed EDLC model for person identification with the recent approaches is presented in Table 5. The classifiers of the suggested EDLC model have outperformed the more modern methods in the tabular representation.

**Table 5:** Comparison of the EDLC model for person identification with current leading approaches.

Data set	Year	Methods	Accuracy (%)
UBIPr	2019	OCLBCP dual-stream CNN^24^	91.28
	2021	CNN + HOG^35^	93.83
	2022	Region-specific and sub-image-based neighbor gradient model^66^	92.32
	2022	LDA-CNN^64^	99.17
	2023	Vision transformer-based model^36^	98.18
	2023	VGG19^65^	98.65
		**Proposed**—**SSA-RTNet**	99.8
		**Proposed**—**PHCSN**	99.6
		**Proposed**—**DAA-CNN**	99.20
UFPR	2022	LDA-CNN^64^	97.68
		**Proposed**—**SSA-RTNet**	99.67
		**Proposed**—**PHCSN**	99.24
		**Proposed**—**DAA-CNN**	97.77

### Analysis in terms of gender classification

In this sub-section, the implications of the proposed model are examined for different evaluation metrics.

**Fig. 11:**
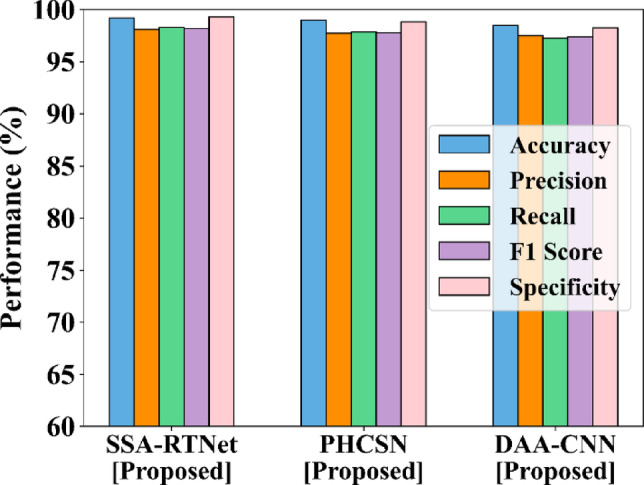
Comparison of gender classification using the UBIPr dataset.

**Fig. 12:**
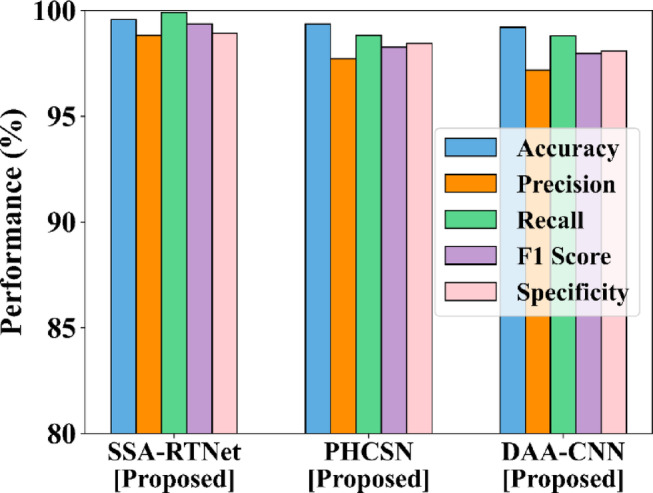
Comparison of gender classification using the UFPR dataset.

Figures 11 and 12 provide the comparison of gender classification using the UBIPR dataset and UFPR dataset for the proposed SSA-RTNet, PHCSN, and DAA-CNN. It appears from the graphical representation that the suggested classifiers have effectively predicted the gender of a person depending on the input data.

The proposed classifier can automatically learn the features of the periocular images and capture significant patterns that indicate gender. Thereby, it is obvious that the proposed method is appropriate for person identification as well as gender classification.

The comparison of the outlined EDLC model for gender classification is presented in Table 6. The classifiers of the suggested EDLC model have performed better in the tabular representation with respect to accuracy, precision, recall, F1-score, and specificity.

**Table 6:** Comparison of the EDLC model for gender classification for various performance metrics.

Metrics (%)	UBIPr Dataset		
	SSATNet	PHCSN	DAA-CNN
Accuracy	99.4	99.28	98.80
Precision	99.64	98.55	98.05
Recall	99.59	98.27	97.36
F1-score	99.59	98.41	97.71
Specificity	99.88	99.5	98.19

Metrics (%)	UFPR dataset		
	SSATNet	PHCSN	DAA-CNN
Accuracy	99.68	99.37	98.50
Precision	98.83	98.32	97.48
Recall	99.91	98.83	97.25
F1-score	99.37	98.57	97.37
Specificity	98.91	98.44	98.25

**Fig. 13:**
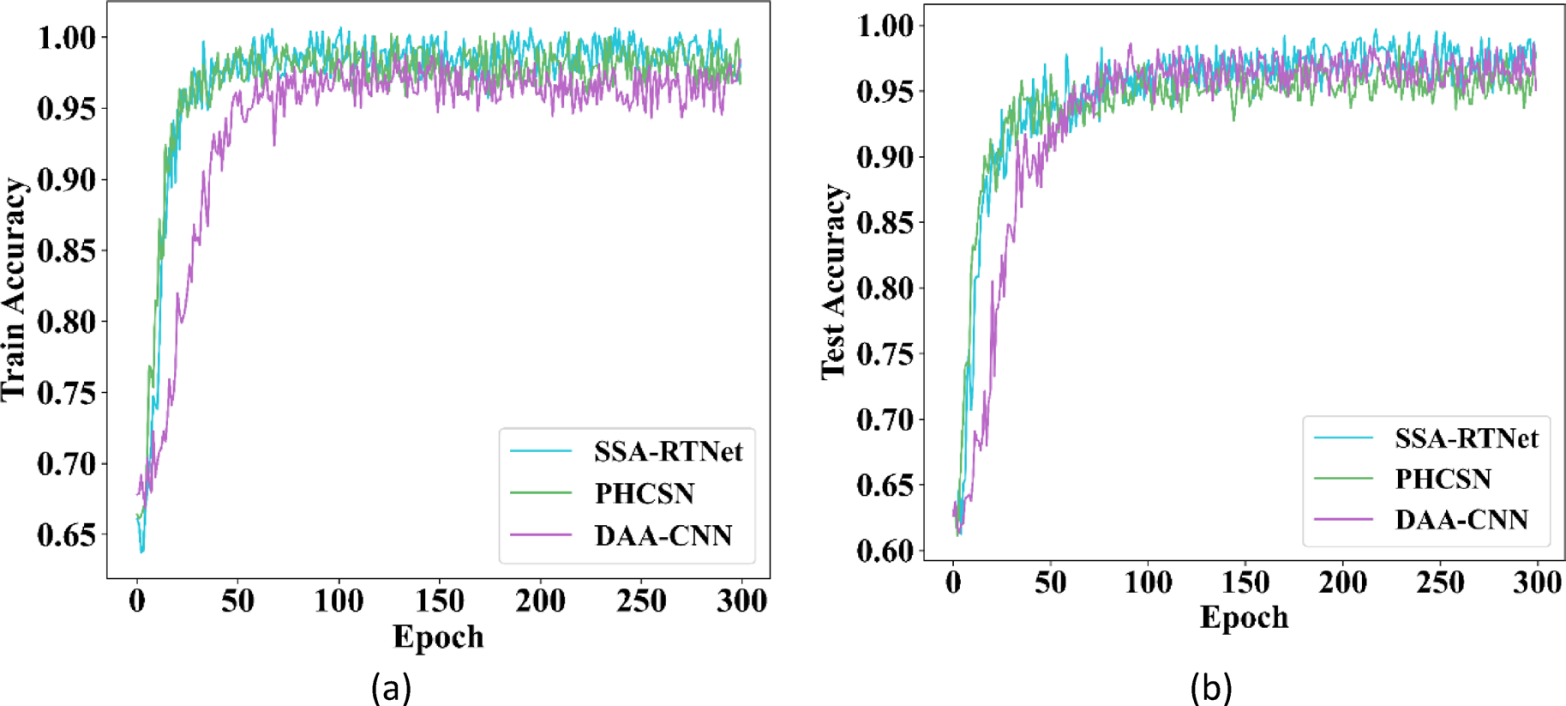
Training and testing accuracy for GC using UBIPr dataset (**a**) Training accuracy (**b**) Testing accuracy.

**Fig. 14:**
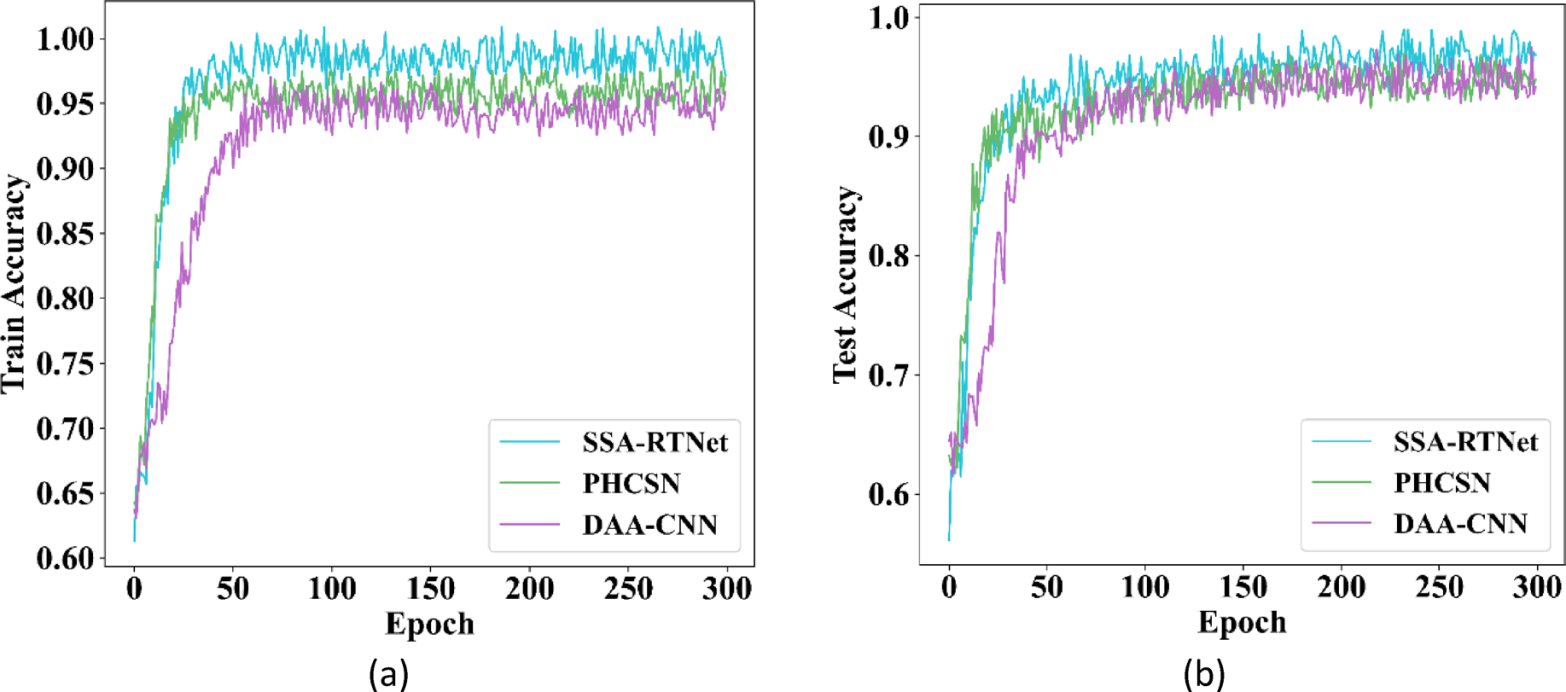
Training and testing accuracy for GC using UFPR dataset (**a**) Training accuracy (**b**) Testing accuracy.

The training and testing accuracy for gender classification for UBIPr and UFPR datasets is furnished in Fig. 13 as well as Fig. 14. From the graphical representation, the attained training accuracy implies, how better the proposed method determined the gender of a person from the train subset. Meanwhile, the testing accuracy clearly indicated the capability of the model to detect gender precisely.

**Fig. 15:**
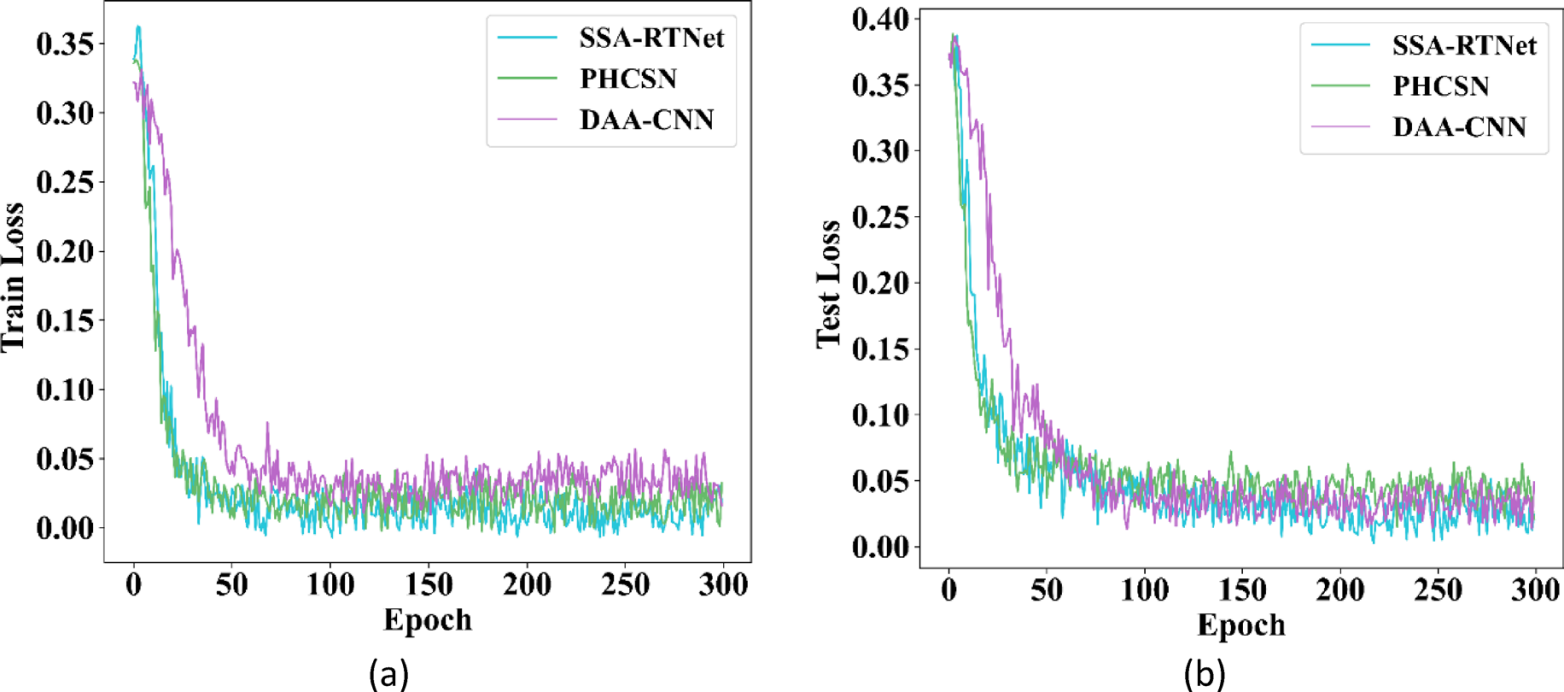
Training and testing loss for GC using UBIPr dataset (**a**) Training loss (**b**) Testing loss.

**Fig. 16:**
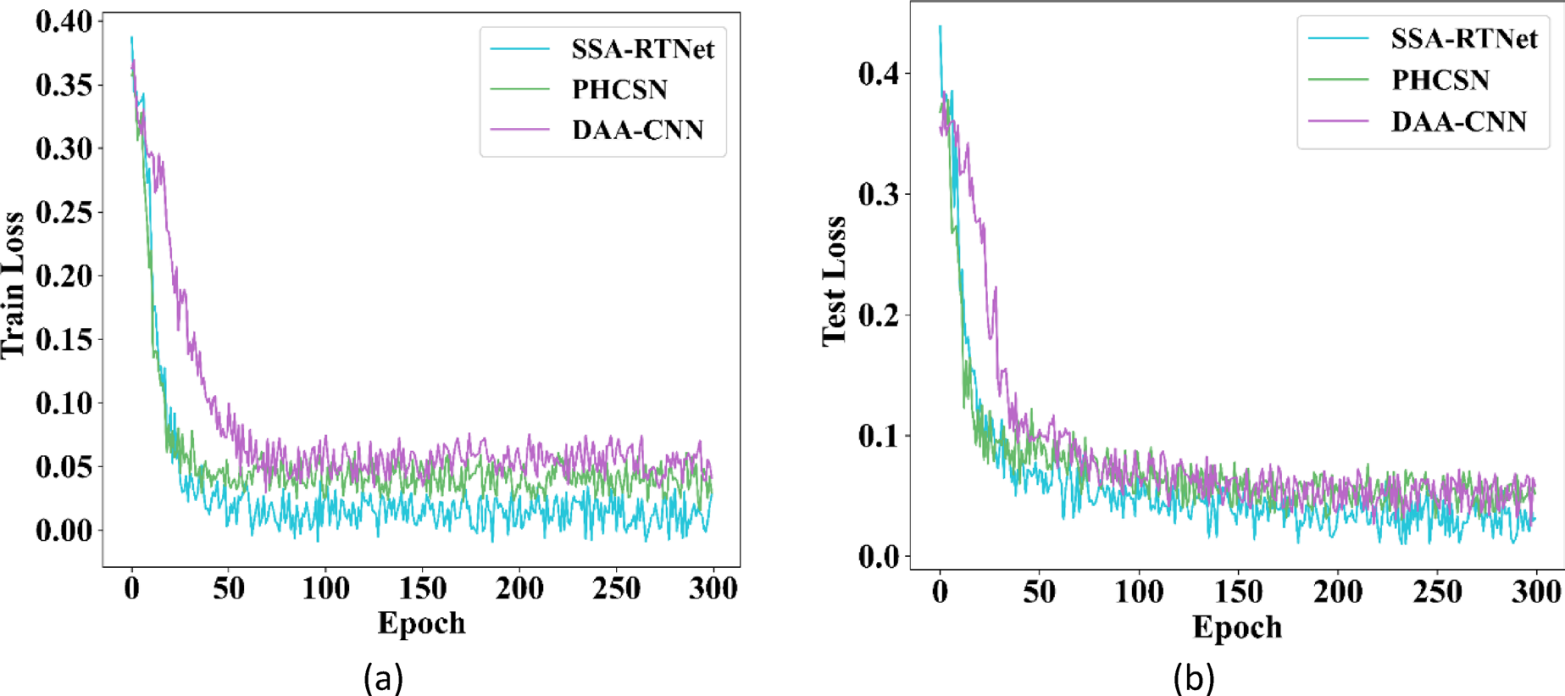
Training and testing loss for GC using UFPR dataset (**a**) Training loss (**b**) Testing loss.

The training and testing loss of gender classification for the UBIPr and UFPR datasets is provided in Figs. 15 and 16. In the graphical depiction; it is observed that the suggested classifiers have attained minimum loss for the training and testing sets.

#### Comparison of gender classification with existing methods

In this subsection, the implementation of the EDLC model is investigated with state-of-the-art methods on unconstrained datasets such as UBIPr and UFPR in terms of accuracy for GC.

The comparison of the suggested EDLC model for GC with the recent approaches is presented in Table 7. It is evident from the tabular representation that the EDLC model surpassed the current ones with regard to gender classification accuracy.

**Table 7:** Comparison of the proposed EDLC model for Gender Classification contrasted with the latest methodologies.

Dataset	Year	Methods	Accuracy (%)
UBIPr	2021	CNN + HOG^35^	95.00
	2023	Vision transformer-based model^36^	99.13
		**Proposed**—**SSA-RTNet**	99.4
		**Proposed**—**PHCSN**	99.28
		**Proposed**—**DAA-CNN**	98.50
UFPR	2023	PeriGender^67^	94.90
		**Proposed**—**SSA-RTNet**	99.68
		**Proposed**—**PHCSN**	99.37
		**Proposed**—**DAA-CNN**	98.80

### Convergence analysis

In this section, convergence analysis is used to confirm the efficacy of ACoOA in the suggested EDLC model. To fine-tune parameters, the convergence curve gives a sharper view of ACoOA’s exploratory as well as leveraging character.

**Fig. 17:**
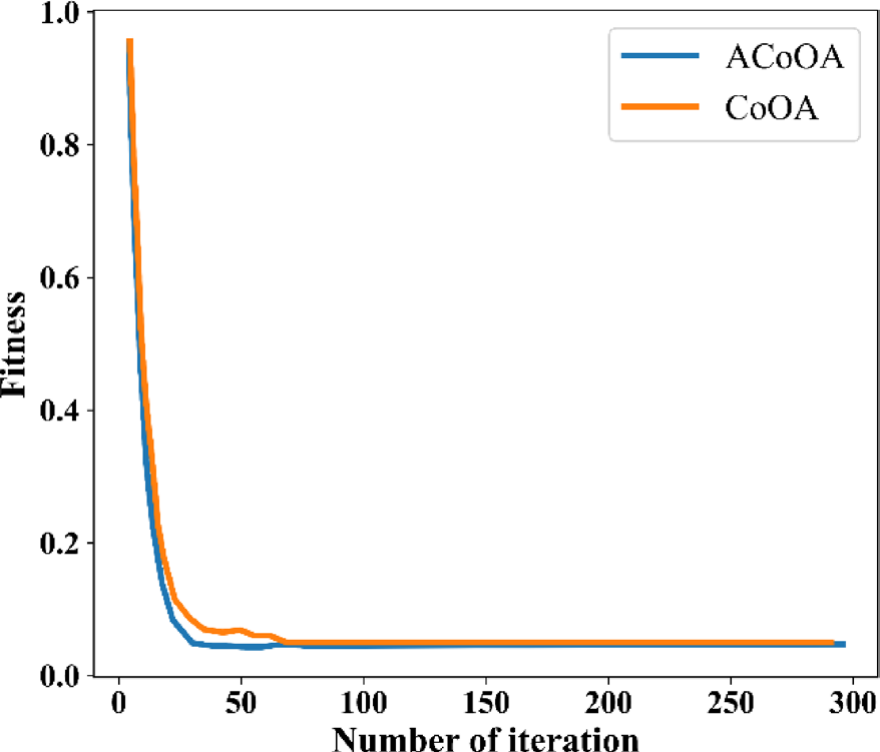
Convergence analysis.

The convergence curve across different optimization procedures is indicated in Fig. 17. The ACoOA and traditional CoOA are contrasted, as shown in the picture. Although ACoOA converges a bit later than regular CoOA in the first iteration, it makes up for it with superior objective function final values.

The reason for the improvement is the utilization of a chaotic tent map to initialize search agents (solution) at the initialization phase of CoOA. This corroborates enhancing the utilization ability together with evading the local optimum problem.

Moreover, the ACoOA improves classification performance by significantly increasing fitness performance and minimizing classification errors. Furthermore, the diagrammatic exemplification indicates that the proposed method necessitates fewer iterations as compared with the existing CoOA. The existent CoOA obliges roughly 40 iterations to optimize the parameters competently. The proposed method obliges merely 30 iterations for maximizing the hyper-parameters of the proposed network models.

Furthermore, during the search iteration, ACoOA was able to strike a balance between exploration and exploitation. Therefore, it is demonstrated by the findings that the suggested ACoOA is better and more capable of avoiding the local optimum problem while increasing the classification percentage.

### Ablation study

The section reveals a number of ablation investigations to examine the resilience of every element of the EDLC model. To investigate every aspect by conducting ablation studies, the EDLC model has been split into four unique categories: Module-E, Module-F, Module-G, and Module-H. Module-E establishes the EDLC model without any pre-processing, Module-F discusses without ROI localization, Module-G indicates without feature extraction, and Module-H emphasizes on without doing appropriate parameter tuning with regard to ACoOA. Table 8 displays the EDLC model’s accuracy-based effectiveness for person recognition and gender classification throughout the ablation studies. Based on each module, Module-E achieves lower performance since pre-processing with image resizing, min–max normalization, and CTri-LGF are not considered. Bypassing the pre-processing, the EDLC model is unable to perform better in person identification and gender classification. In Module F, ROI localization to obtain the interested region is eradicated. Module-G indicates the EDLC by obliterating the Laplacian transform for extracting features. The use of the Laplacian transform emphasizes textures, edges, and structural characteristics, and improves feature extraction, which is significant for the proposed model’s performance. With the Laplacian transform, the EDLC model accomplished maximum accuracy of 99.8%, 99.6%, and 99.20 for SSA-RTNet, PHCSN, and DAA-CNN using the UBIPr dataset. Similarly, using the UFPR dataset, the EDLC model achieved its best accuracy of 99.67%, 99.24%, and 97.77%. On the other hand, when considering gender classification, the EDLC model also reached maximum accuracy of 99.4%, 99.28%, and 98.5%; and 99.68%, 99.37%, and 98.80% for SSA-RTNet, PHCSN, and DAA-CNN using UBIPr and UFPR datasets by including the Laplacian transform. But, without the Laplacian transform, the model experienced a decline in its capability to acquire these high-frequency features, which is vital for person identification and gender classification. This would have ensued in minimized classification accuracy and overall performance, as shown in Table 8. Accordingly, Module-H revealed that parameter tuning through ACoOA is necessary for minimizing loss and getting better performance. Overall, from the ablation study, we can conclude that every element in the EDLC model is important in increasing the performance of person identification and gender classification.

**Table 8:** Performance achieved by the EDLC model in terms of accuracy (%) through the ablation study.

Dataset	Person identification				
	Classifier	Module E	Module F	Module G	Module H
UBIPr	SSA-TNet	91.46	87.34	89.65	95.05
	PHCSN	91.26	87.16	89.36	94.83
	DAA-CNN	91.02	87.09	89.16	94.64
UFPR	SSA-TNet	91.16	87.03	89.48	94.93
	PHCSN	89.63	85.47	87.58	92.37
	DAA-CNN	89.48	85.79	87.89	92.75

Dataset	Gender classification				
	Classifier	Module E	Module F	Module G	Module H
UBIPr	SSA-TNet	90.99	86.83	88.94	94.73
	PHCSN	90.78	86.63	88.73	94.56
	DAA-CNN	90.71	86.53	88.60	94.47
UFPR	SSA-TNet	90.83	86.83	88.96	94.69
	PHCSN	90.97	86.89	88.84	94.38
	DAA-CNN	90.79	86.74	88.73	94.24

### Error analysis

**Fig. 18:**
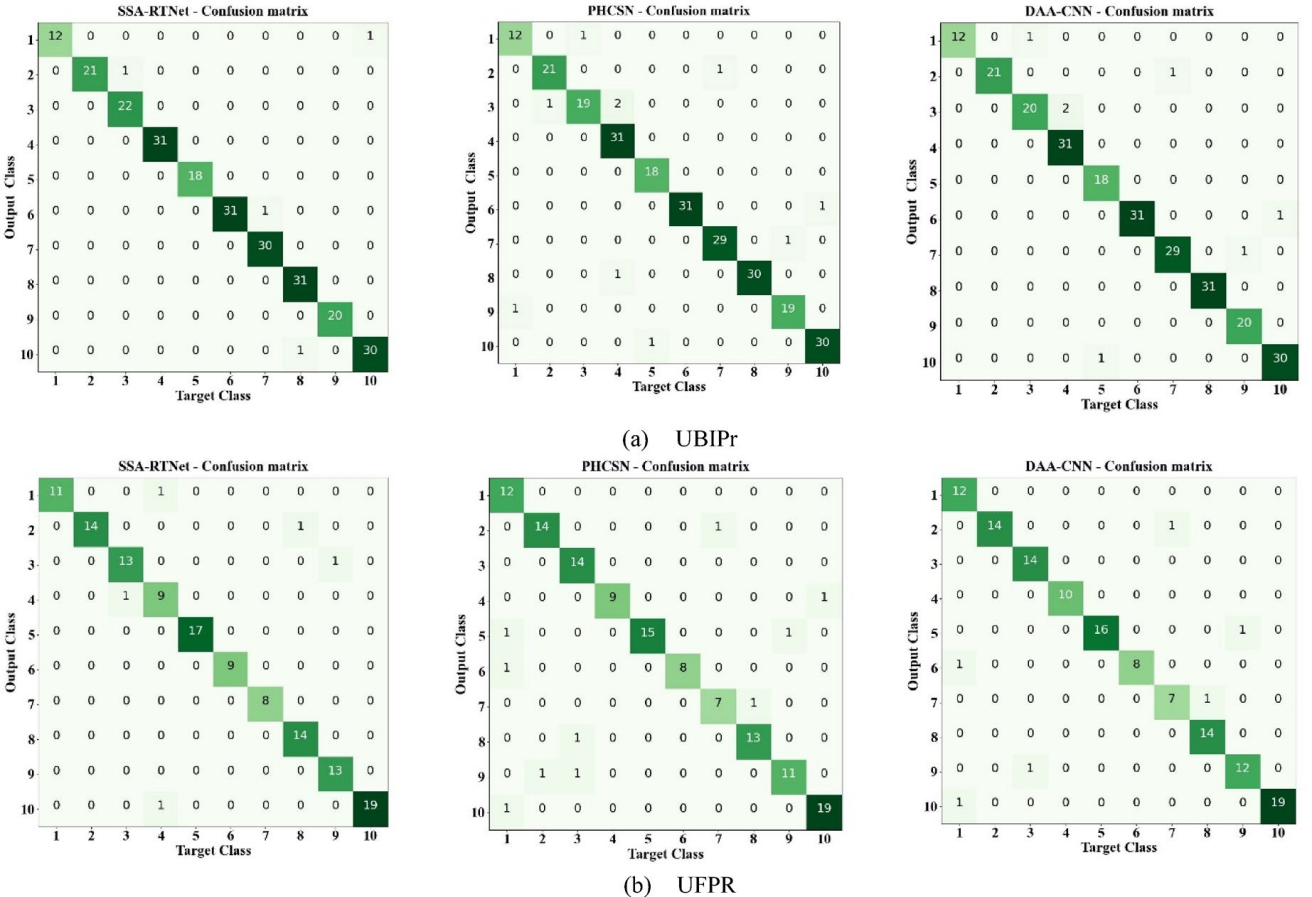
Confusion matrix for person identification (**a**) UBIPr (**b**) UFPR.

A confusion matrix is deliberated as a significant tool for analyzing errors in classification models since it offers a clear representation of correct and incorrect detections across various classes. It assists in determining misclassification patterns, exposing which classes are often confused owing to data imbalance, similarities in features, or external factors like lighting variations, occlusions, or resolution changes. Thereby, in this section, the detailed error analysis of the EDLC model is performed through confusion matrices. A total of 10 classes (Person 1–Person 10 different person periocular images) are considered for person identification to validate the detection efficiency of the proposed classifiers. In each class, a single person’s periocular image is available with different conditions such as occlusions and variations. Figure 18 shows the confusion matrix of various classifiers of the EDLC model such as SSA-TNet, PHCSN, and DAA-CNN that uniquely identifies each person using UBIPr and UFPR datasets. It is evident from the confusion matrix that the EDLC approach reduced the rate of inaccurate predictions for the provided images and correctly identified the class labels. Additionally, the diagonally represented values in the confusion matrix graphic illustrate the quantity of correctly predicted values for the samples. On the other hand, the remaining values describe the incorrectly identified values. Some classes have attained perfect accuracy with zero false predictions. The remaining few classes yielded better performance with fewer incorrect predictions.

**Fig. 19:**
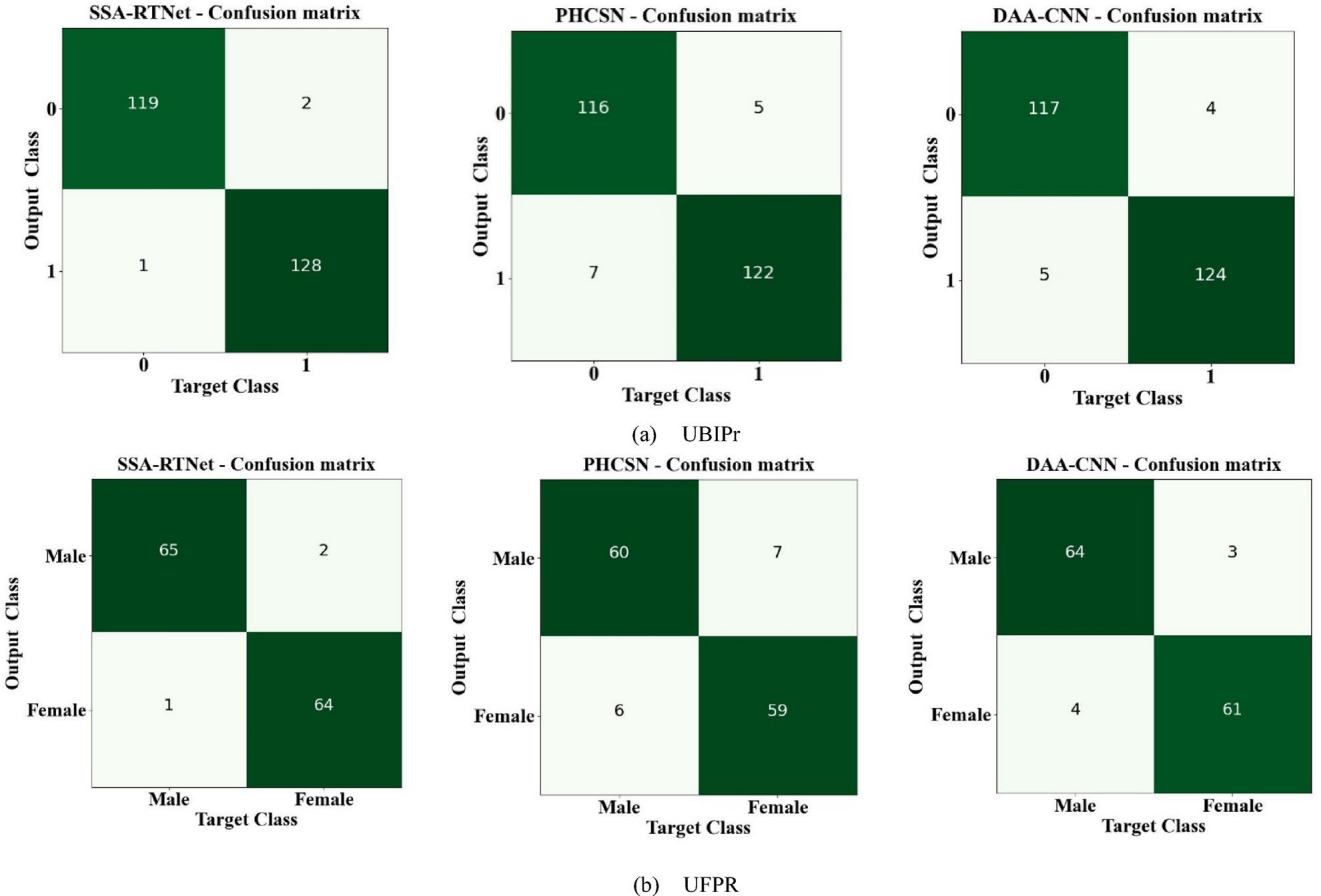
Confusion matrix for gender classification (**a**) UBIPr (**b**) UFPR.

Figure 19 envisages the confusion matrix of EDLC models such as SSA-TNet, PHCSN, and DAA-CNN for gender classification. Here, the confusion matrix clearly symbolized how different classifiers introduced in the EDLC model distinguishes between the person’s gender as male or female based on the periocular features. From the graphical representation, it is perceived that the classifiers of the proposed EDLC framework have determined the class labels (male and female) of an individual with an improved detection rate.

### Statistical analysis

The non-parametric analysis of EDLC and current models on UBIPr and UFPR datasets is presented in this section. Analysis of variance (ANOVA) is a method for analyzing experimental data that uses one or more classification variables to estimate one or more response variables under various situations. In the proposed method, an ANOVA test is performed to analyze the evaluation of the EDLC model. Table 9 lists the results of the ANOVA test for EDLC models such as SSA-TNet, PHCSN, and DAA-CNN and prevailing SNN, SAE, and DBN models according to accuracy metrics. The ANOVA test’s *p*-value must be fewer than 0.05 in order to reject the null hypothesis. Table 9 indicates that the p-value is fewer than 0.05. Thus, the ANOVA test findings disproved the null hypothesis.

**Table 9:** Anova test for accuracy results obtained using proposed and existing models.

Dataset	Person identification						
	SNN	SAE	DBN	BiLSTM	SSA-TNet	PHCSN	DAA-CNN
UBIPr	0.0512	0.0489	0.0422	0.0401	0.0223	0.0137	0.0022
UFPR	0.0421	0.0374	0.562	0.503	0.0341	0.0141	0.0031

Dataset	Gender classification						
	SNN	SAE	DBN	BiLSTM	SSA-TNet	PHCSN	DAA-CNN
UBIPr	0.0515	0.0464	0.0417	0.0313	0.0201	0.0156	0.0031
UFPR	0.0465	0.0686	0.553	0.513	0.0267	0.0152	0.0024

## Discussion

In the field of CV and artificial intelligence, person identification and gender classification are two tasks that are envisioned to identify persons in images or videos and discover their gender. In order to complete these tasks, machine learning algorithms that have been trained on larger datasets of labeled images enable the system to learn the features and patterns that are indicative of particular persons or genders. However, while learning discriminative features, deep learning models have established superior performance. Thereby, the proposed work has suggested dissimilar EDLC to instinctively understand incoming data’s hierarchical representations, which can recognize and classify gender as well as identify complicated patterns and variations. The proposed EDLC model enhanced the robustness of the classifiers such as DAA-CNN, SSA-RTNet, and PHCSN by providing an adaptive optimizer that supports to prevention of over-fitting and increases generalization performance. Also, the inclusion of the Chebyshev map into CoOA maximizes the robustness and encourages the classifiers to learn the features.

Moreover, the EDLC model can optimize both feature representation learning and classification simultaneously for better performance. The capacity of the EDLC model to generalize effectively across many datasets is recognized. Since the EDLC model adjusts to a wide range of data distributions, it is more reliable in real-world circumstances where the data can differ in terms of lighting, quality, position, and other factors. In order to reach competitive performance, the EDLC model is built by combining developments in optimization algorithms and architectural design. Besides, the proposed EDLC model is robust and advantageous since it can automatically learn discriminative features and considerably perform better against the state-of-the-art methods of person identification as well as gender classification tasks. Overall, the proposed EDLC model has a positive impact on person identification and gender classification. These include greater accuracy, increased robustness, flexibility to different tasks, advances in DL methods, and the possibility of hybrid approaches.

## Conclusion

This paper proposed a robust system using EDLC for person identification and gender classification using periocular images. The EDLC model comprises five essential phases: image acquisition, pre-processing, region-of-interest (ROI) localization, feature extraction, and classification. By utilizing rich biometric features from the periocular region, the EDLC model has proven superior performance across different performance indicators including precision, F1-score, specificity, recall, and accuracy, on UBIPr and UFPR-Periocular datasets. The proposed model contributes to the field by effectively addressing challenges in periocular-based identification, offering a reliable alternative to traditional methods. Additionally, the proposed EDLC reveals a significant enhancement in accuracy, making valuable insights into the field of computer vision and biometric authentication. Even attaining supremacy, the proposed EDLC implicates dimensionality issues and can result in significant information loss due to the ignorance of feature selection, which tends to increase complexity. In the future, a novel hybrid optimization technique can be focused on choosing the most discriminative features and introducing advanced knowledge distillation-based methods to improve the performance of classification, lessen misclassification, and minimize the complexity.

## Data Availability

The datasets analysed during the current study are available in the UBIPR website, http://iris.di.ubi.pt/ubipr.html and UFPR website, https://web.inf.ufpr.br/vri/databases/ufpr-periocular/.
